# Nanostructured Boron‐Doped Ultra‐Nanocrystalline Diamond Micro‐Pyramids: Efficient Electrochemical Supercapacitors

**DOI:** 10.1002/smll.202407514

**Published:** 2024-12-15

**Authors:** Shradha Suman, Dhananjay Kumar Sharma, Ondrej Szabo, Benadict Rakesh, Marian Marton, Marian Vojs, Kamatchi Jothiramalingam Sankaran, Alexander Kromka

**Affiliations:** ^1^ CSIR‐Institute of Minerals and Materials Technology Bhubaneswar 751013 India; ^2^ Academy of Scientific and Innovative Research (AcSIR) Ghaziabad 201002 India; ^3^ Institute of Physics of the Czech Academy of Sciences Prague 16200 Czech Republic; ^4^ Institute of Electronics and Photonics Slovak University of Technology Bratislava 81219 Slovakia

**Keywords:** boron, graphite, micro‐pyramids, nanorods, supercapacitors, ultra‐nanocrystalline diamond

## Abstract

The miniaturization of electrochemical supercapacitors (EC‐SCs) requires electrode materials that are both durable and efficient. Boron‐doped diamond (BDD) films are an ideal choice for EC‐SC due to their durability and exceptional electrochemical performance. In this study, nanostructured boron‐doped ultra‐nanocrystalline diamonds (NBUNCD) are fabricated on Si micro‐pyramids (Si_P_) using a simple reactive ion etching (RIE) process. During the etching process, the high aspect ratio and the induction of *sp* ^2^ graphite in these nanorod electrodes achieved a maximum specific capacitance of 53.7 mF cm^−2^ at a current density of 2.54 mA cm^−2^, with a 95.5% retention after 5000 cycles. Additionally, the energy density reached 54.06 µW h cm^−2^ at a power density of 0.25 µW cm^−2^. A symmetric pouch cell using NBUNCD/Si_P_ exhibited a specific capacitance of 0.23 mF cm^−2^ at 20 µA cm^−2^, an energy density of 31.98 µW h cm^−2^, and a power density of 0.91 µW cm^−2^. These superior EC properties highlight NBUNCD/Si_P_’s potential for advancing miniaturized supercapacitors with high capacitance retention, cycle stability, and energy density.

## Graphical abstract

Nanostructured diamond electrodes fabricated via substrate structuring and reactive ion etching demonstrate markedly enhanced specific surface area, specific capacitance, and electrochemical stability, advancing their potential for high‐performance energy storage applications.

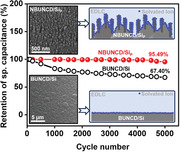

## 1 Introduction

In the generation of relentless pursuit of innovation, miniaturized devices stand poised on the pillars of efficiency, durability, and safety in energy storage. Intense research has been conducted to address the limitations in power densities and the lifetime of current rechargeable batteries, which aligns with ongoing global efforts.^[^ ^1^ ^]^ Traditionally, batteries harness high energy density via a slow reversible electrochemical reaction. In contrast, EC‐SCs store energy at the electrode‐electrolyte interface through electrostatic adsorption/desorption and/or a fast Faradaic mechanism. This allows them to deliver a high amount of energy in a comparatively short time, that, high power density, faster charging and discharging rates, and significant lifecycle stability.^[^ ^2^ ^]^ The unique capability of the EC‐SCs to near‐instantaneous power discharge renders them ideal for applications requiring uninterrupted power delivery. Notably, the EC‐SCs are currently well‐suited for devices requiring short bursts of high power, as is preferred in hybrid automobiles such as buses, trains, elevators, and cranes for regenerative braking and burst mode power delivery. Furthermore, the EC‐SCs are extensively utilized in the realm of medical equipment, as well as short‐term energy storage devices.^[^ ^3^ ^]^

The demand for energy storage systems has redirected attention towards finding solutions to the drawbacks of both batteries (slow rates and low power density) and SCs (relatively low energy density). The remedy to this problem is found by exploring and exploiting electrode materials.^[^ ^4^, ^5^ ^]^ The energy density of the SC can be enhanced by improving the specific capacitance (C_S_) and/or the operating voltage (V) of the system (as E = 1/2 CV^2^).^[^ ^1^ ^]^ To enhance the C_S_, various electrode materials such as transition metal oxides, carbon allotropes (graphene, CNTs, fullerenes, activated carbons), and their composite materials have been investigated to improve EC performance by increasing specific surface area, porosity, and conductivity.^[^ ^3^ ^]^ The enhancement of working potential (V) can be achieved through three routes: i) the use of organic electrolytes in symmetric SCs, ii) the design of asymmetric SCs, and iii) the selection of larger electrochemical potential window samples.^[^ ^6^, ^7^ ^]^ However, the second method has several inherent drawbacks, including decreased conductivity, reduced safety, and environmental unfriendliness. Therefore, the optimization of electrode material was chosen as a viable approach. However, the characteristics of high‐performance SC electrodes are i) high surface area (as the charge is stored at the surface of the electrode), ii) electronic and ionic conductivity (provides efficient electron pathways for charge transport), and iii) mechanical and chemical stability (as phase change and side reactions of the active materials are the major cause of cycle instability).^[^ ^8^ ^]^

Diamond, the known hardest, chemically inert, anti‐fouling, and corrosion‐resistant material, can be employed for the electrode materials in EC‐SC to overcome stability issues. The diamond samples with a high defect density are stable in harsh environments, such as acidic electrolytes and higher operating temperatures.^[^ ^5^ ^]^ However, they exhibit rich surface chemistry, indicating that diamond electrodes can be used to develop novel types of SCs.^[^ ^9^ ^]^ Other carbon‐based electrode materials in a redox‐active electrolyte, such as carbon nanotubes, show 5–9 µF cm^−2^ specific capacitance,^[^ ^10^ ^]^ and graphene shows 10–40 µF cm^−2^,^[^ ^11^ ^]^ whereas BDD shows 234 – 300 µF cm^−2^.^[^ ^12^, ^13^ ^]^ The BDD film is a promising electrode in the EC‐SC because it possesses a wide electrochemical working potential in all types of electrolytes, along with high mechanical hardness, chemical inertness, and thermal stability. However, the bulky BDD film has large micron‐sized grains, resulting in low capacitance values and high interfacial impedance, limiting its performance as EC electrodes.^[^ ^14^, ^15^ ^]^ Therefore, several attempts have been made to reduce the grain sizes of diamonds, and boron‐doped ultra‐nanocrystalline diamond (BUNCD) has been fabricated via chemical vapor deposition (CVD) methods with a diverse set of favorable properties, making BUNCD suitable for use in EC devices.^[^ ^16^, ^17^ ^]^ The BUNCD inherits all the robust qualities of diamonds but in smaller grain sizes, exceeding the physical and chemical properties of conventional diamonds.^[^ ^18^, ^19^ ^]^ The decrease in diamond grain sizes increases the number of grain boundaries, thereby enhancing the *sp^2^*/*sp^3^* ratio in the material along with the content of other forms of non‐diamond carbon, such as amorphous carbon and hydrocarbons.^[^ ^18^ ^]^ The BUNCD films possess remarkable chemical inertness and substantial stability, even at high current densities and potentials. Moreover, the BUNCD film has a wide potential window of ≈3.2 V in aqueous and 4.6 V in organic solutions, making it an exceptional choice for EC applications.^[^ ^20^ ^]^

In order to further augment the EC‐SC performance, efforts were directed towards amplifying the surface area. Two standard methods for achieving this are substrate structuring and the fabrication of diamond nanostructures. Before the diamond CVD, texturing the substrate enhances the electrical conductivity of thin films and provides a high aspect ratio template.^[^ ^21^ ^]^ Over the past decades, researchers have successfully achieved substrate structuring through chemical vapor etching, laser ablation, lithography, RIE, and thermal evaporation/deposition techniques.^[^ ^22^, ^23^, ^24^, ^25^, ^26^ ^]^ These methods have yielded substrates with nanowires, erect or inverted pyramids, and more. However, these approaches rely on sophisticated advanced equipment and complex processing steps, which makes them both time‐consuming and expensive. To overcome these limitations, using alkali wet‐etching of substrates provides a relatively simple, efficient, and cost‐effective approach for fabricating structured substrates.^[^ ^21^, ^27^, ^28^ ^]^ Several etching methods for the diamond post‐growth treatment have been reported as the second approach to enhancing surface area through diamond nanostructuring.^[^ ^29^ ^]^ However, wet etching is ineffective for structuring diamond films due to the extreme hardness of diamonds. RIE has become a preferred method for creating nanostructures on diamond surfaces due to its convenience, cost‐effectiveness, and significant improvement in areal densities and reproducibility.^[^ ^30^, ^31^ ^]^

This work describes the fabrication of NBUNCD over a Si_P_ substrate. The Au mask‐assisted RIE process is employed to create nanostructures on BUNCD films. The resulting NBUNCD on Si_P_ was employed as an EC‐SC electrode and exhibited a remarkable current response and high stability. The electrode was characterized using cyclic voltammetry (CV) and galvanostatic charging‐discharging (GCD) techniques to examine the enhanced EC characteristics. Electrochemical impedance spectroscopy (EIS) was employed to study the effect of micro‐pyramid structure on the substrate and nanostructuring of BUNCD on the EC performance of the electrode. Furthermore, this study estimates the specific capacitance, lifecycle stability, energy, and power densities of the electrode, and power densities. The results show that the EC‐SC performance of the NBUNCD/Si_P_ electrode is enhanced compared to other nanostructured electrodes. This enhancement is attributed to the high aspect ratio and the presence of sp^2^ graphite in the electrode.

## 2 Experimental Section

### 2.1 Fabrication of Si Micro‐Pyramids

**Figure 1:**
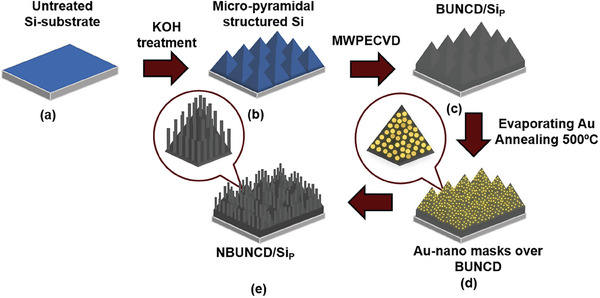
Schematic representation of the fabrication of nanostructured BUNCD on Si micro‐pyramids.

**Figure** **1** shows a schematic representation of the fabrication of NBUNCD on Si_p_. The Si substrate was structured using the alkali etching technique to engineer the Si_p_.^[^ ^32^, ^33^ ^]^ First, (100) Si substrates (Figure 1a) were cleaned in acetone, isopropyl alcohol, and demineralized water and then rinsed in a solution of KOH, isopropyl alcohol, and demineralized water (1:6:55) at 90 °C for 45 min.^[^ ^34^ ^]^ This process resulted in the formation of micro‐pyramidal structures over the Si substrate (Figure 1b). The structures were formed due to the preferential anisotropic etching along Si crystal planes.^[^ ^35^, ^36^ ^]^

### 2.2 Preparation of BUNCD Films

The BUNCD films were fabricated on Si_P_ substrates using the LA MW CVD reactor (SCIA cube 300). First, the substrates were subjected to ultrasonic nucleation in a suspension of nanodiamond powder with a particle size of ≈5 nm in deionized water. Trimethyl borate (TMBT) was employed as a carbon, boron, and oxygen source for film growth.^[^ ^37^ ^]^ The 30‐h growth was conducted in a gas mixture of H_2_, TMBT, and CO_2_ with a CO_2_ to H_2_ ratio of 0.2%. The flow rate of evaporated TMBT was maintained at 4%, resulting in a B/C ratio of 328 000 ppm. The substrate temperature was maintained at 600 °C, and the pressure was kept at 30 Pa for forming BUNCD films on Si_P_ substrates, designated as BUNCD/Si_P_ (Figure 1c). For comparison, the BUNCD films were also grown on planar Si substrates, designated as BUNCD/Si.

### 2.3 Fabrication of NBUNCD on Si Micro‐Pyramids

First, an 8 nm layer of gold (Au) was evaporated over the BUNCD/Si_P_ film. The Au‐coated BUNCD/Si_P_ film underwent heat treatment in an H_2_‐based microwave plasma for 10 min at 500 °C. This step resulted in the formation of self‐organized nano‐droplets of Au, arranged in an array over the surface of BUNCD/Si_P_ (Figure 1d).^[^ ^38^ ^]^ The Au‐masked diamond films underwent an etching process using a standard capacitively coupled plasma system (Phantom III, Trion Technology) with a mixture of oxygen and 5% tetrafluoromethane (O_2_/CF_4_ – 60/3 sccm) to fabricate 1D nanorods on BUNCD films. The pressure of 150 mTorr and the RF power of 150 W was maintained throughout the experiments. The etching process was carried out for 6 min. Following the RIE process, the remaining Au nano‐droplets were removed by a standard wet chemical etching process (HNO_3_:HCl at 1:3 n/n). The obtained samples were designated as NBUNCD/Si_P_ (Figure 1e).

### 2.4 Materials Characteristics

The morphological analysis of the BUNCD films was examined through field emission scanning electron microscopy (FESEM, Tescan MAIA3). Bruker D8 ADVANCE diffractometer with X‐ray tube with Cu anode operating at 1.6 kW (40 kV/40 mA) was used to analyze BUNCD layers. All X‐ray measurements were performed in parallel beam geometry with a parabolic Goebel mirror in the primary beam. The XRD patterns were measured in the grazing incidence set‐up. Measurements were performed for the value of incidence angle *α*   =   0.3°, in the angular range 10°–100° with step size 0.025°. A parallel plate collimator with angular acceptance of 0.2° was inserted in the diffracted beam. The bonding characteristics of the samples were investigated using micro‐Raman spectroscopy (Renishaw, with λ = 532 nm) and XPS (Phoibos 150, Specs). AFM topography was characterized by an NTEGRA Prima system (NT‐MDT) using BudgetSensors probes – Multi75E‐G. Data processing and analysis were done using Gwyddion software.

### 2.5 Performance of Supercapacitors

A portable PalmSens potentiostat/galvanostat, version 4, controlled by PSTrace 5 software, was utilized for the EC measurements of BUNCD films. All evaluations of the EC‐SC performance of BUNCD samples as working electrodes were conducted employing a three‐electrode configuration, consisting of a reference electrode: Ag/AgCl electrode (immersed in saturated 3 m KCl) and a counter electrode: Pt wire. Two types of electrolytes were employed for the EC measurements: an inert aqueous electrolyte, sodium sulfate (1 m Na_2_SO_4_) and a redox‐active electrolyte, ferro/ferri cyanide (0.05 m [Fe(CN)_6_]^3−/4−^ in 1 m Na_2_SO_4_) in the same cell set‐up to study the electrical double layer capacitance (EDLC) and pseudo capacitance (PC) behavior of the NBUNCD/Si_P_, respectively. The GCD measurements were carried out using the multistep potentiometry mode within the specified voltage range obtained from the CV measurements. EIS was employed to investigate the charge‐transfer kinetics of the electrode. A two‐electrode symmetric supercapacitor was fabricated to evaluate the full‐cell performance, utilizing two NBUNCD/Si_P_ films as electrodes and a 0.35 mm thick Whatman filter paper as the separator. The device employed 3 m KOH solution as the electrolyte. CV, GCD, and a 1000‐cycle stability test of NBUNCD/Si_P_ were conducted. The specific capacitance, energy density, and power density of the symmetric cell were also calculated.

## 3 Results and Discussion

**Figure 2:**
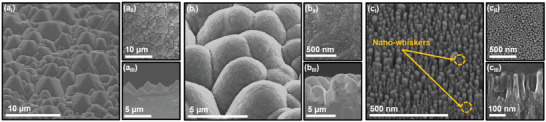
FESEM micrographs a) micro‐pyramidal Si substrate, b) BUNCD/Si_P_, and c) NBUNCD/Si_P_ with (a_I_, b_I_, c_I_) 45° angle view, (a_II_, b_II_, c_II_) plan‐view, and (a_III_, b_III_, c_III_) cross‐sectional view.

The surface morphology FESEM micrographs of the Si_P_ are shown in **Figure** **2**a. The formation of micron‐sized pyramids is distinctly visible in the 45° angle view FESEM micrograph in Figure 2a_I_. The micro‐pyramids of various sizes are randomly distributed throughout the Si substrate. The width of the micro‐pyramids calculated from Figure 2a_II_ varies from 0.5 to 10 µm, and the height ranges between 0.4 to 5.5 µm, calculated from cross‐sectional FESEM Figure 2a_III_.

These structured Si‐substrates were then subjected to the linear antenna microwave plasma enhanced chemical vapor deposition (LA MW CVD) reactor for the diamond deposition, resulting in a uniform coating of BUNCD over the Si_p_ structures. The 45° angle view FESEM micrograph shown in Figure 2b_I_ depicts a uniform coating of BUNCD over Si_P_. The grain sizes for the BUNCD/Si_P_, calculated from the top view FESEM micrograph Figure 2b_II_, range from 20–30 nm with a cauliflower shape.^[^ ^25^ ^]^ Figure 2b_II_ shows a smooth surface with many agglomerated grains, as previously observed for undoped UNCD films grown in Ar‐rich plasma.^[^ ^39^, ^40^, ^41^ ^]^ The cross‐sectional FESEM micrograph analysis from Figure 2b_III_ reveals that the deposition thickness of the BUNCD film is ≈4 µm.

**Figure 3:**
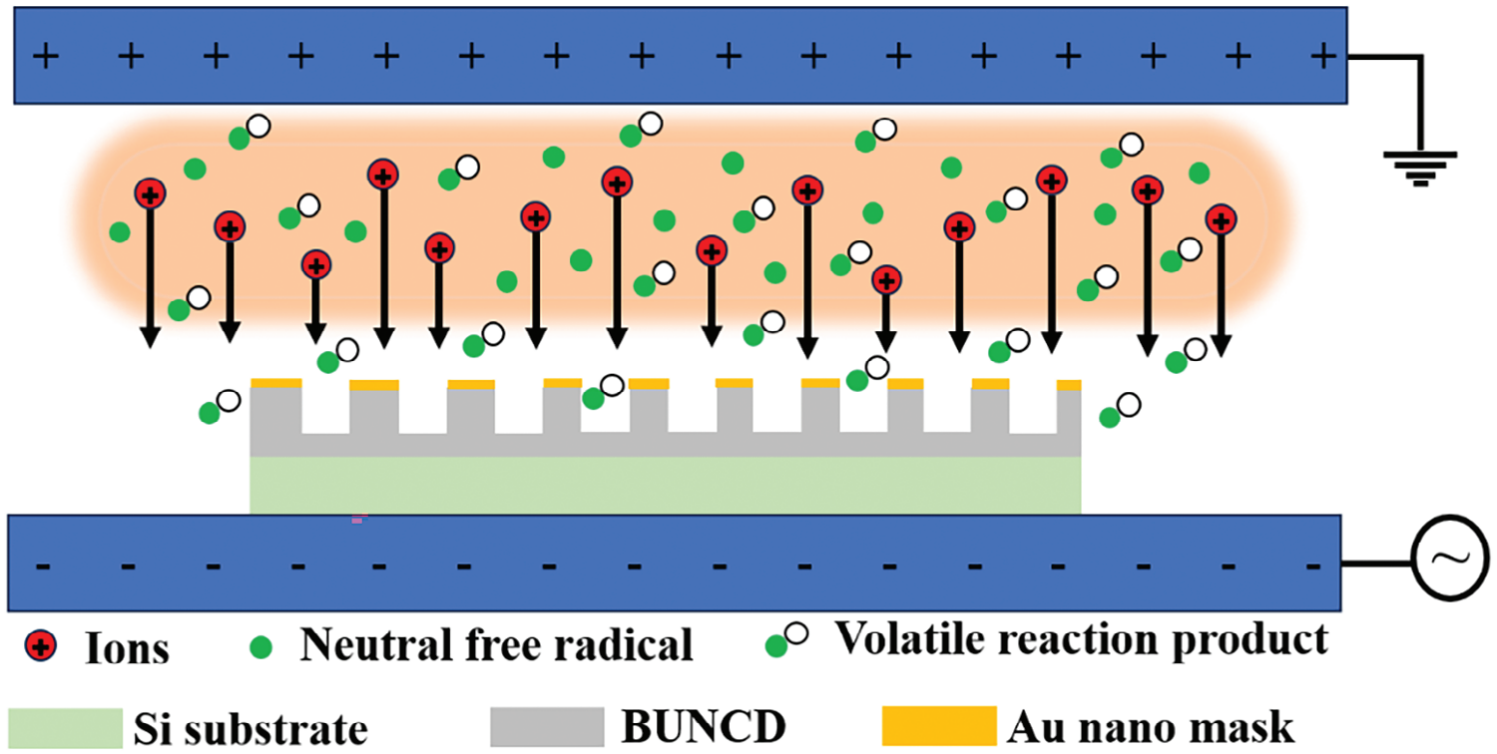
Schematic representation of the mechanism of nanostructure fabrication on BUNCD films using RIE.

The nanostructure fabrication commenced by evaporating an Au‐layer of 8 nm thickness over the BUNCD/Si_P_ film, followed by annealing in microwave plasma at 500 °C, which resulted in self‐organized nano‐droplets of Au on the BUNCD/Si_P_ film. Figure S1 (Supporting Information) shows the top‐view FESEM micrograph of Au nano‐droplets on BUNCD/Si_P_, which were used as the mask; their diameter ranges from 40–65 nm.^[^ ^42^ ^]^ However, clusters of seemingly smaller Au nano‐droplets with diameters ranging from 10–20 nm can also be identified. Due to the structured character of the BUNCD/Si_P_ surface, the smaller Au nano‐droplets are preferentially formed at the foot valleys of the micro‐pyramids. Subsequently, the Au/BUNCD/Si_P_ film was subjected to RIE in O_2_/CF_4_ plasma. The Au nano‐mask‐covered area remained unchanged, whereas the remaining diamond surface was etched out, resulting in vertically aligned nanorods standing upright over the BUNCD/Si_P_ film. Figure 2c_I_ shows the 45° angle view FESEM micrograph of NBUNCD/Si_P_ where a large number of nanorods are distinctly visible with a wide range of tip‐diameter up to 65 nm, as calculated from Figure 2c_II_. The length of the nanorods is ≈220 nm, and the width is ≈40–60 nm, as calculated from Figure 2c_III_. Nano‐whiskers with smaller tip diameters ≈10–20 nm were also observed among the NBUNCD/Si_P_ nanorod arrays. The formation of nano‐whiskers can be attributed to two aspects. First, the diameter of the BUNCD grains is smaller than that of the diameter of the Au‐mask, resulting in the formation of smaller Au nano‐droplets at the valley of the micro‐pyramids, along with an uneven etching of the diamond grains.^[^ ^42^, ^43^ ^]^ Another reason can be the rapid anisotropic nature of RIE.^[^ ^44^, ^45^ ^]^ The high‐energy oxygen and fluorine ions in the plasma accelerate towards the diamond film under the influence of RF bias, as shown in **Figure** **3**.^[^ ^40^, ^41^ ^]^ This disrupts the BUNCD, and some of the carbon interacts with the plasma species, thus enhancing the efficiency of the etching process. The disordered carbon, such as hydrocarbons and/or amorphous carbons, at the grain boundaries gets etched out due to the highly energetic oxygen ions resulting in nano‐whiskers.^[^ ^27^, ^28^ ^]^ The chemical reaction involved in the etching of diamond in the RIE system can be realized via the equation: *C*_(*diamond*)_ + *CF*_4_ + *O*_2_ → *C*_(*accumulated*)_ + *CF_x_* + *CO*, where *C*_(*diamond*)_ is carbon element of etched diamond and *C*_(*accumulated*)_ is the deposition of amorphous carbon over the diamond surface.^[^ ^46^ ^]^ Figure 3 depicts the etching of the unmasked portion of BUNCD/Si_P_ due to the bombardment of highly energetic ions on the diamond surface with an accumulation of carbon and emission of CO. Thus, the conversion of *sp* ^3^‐bonded carbon to *sp* ^2^‐bonded carbon occurs with the formation of BUNCD nanostructures.^[^ ^47^ ^]^ However, due to the presence of Au‐masks and CF_4_, the resulting nanostructures have nearly uniform lengths with smooth surfaces.^[^ ^48^ ^]^

For comparison, BUNCD films were also grown on a polished Si substrate, and the FESEM micrograph of BUNCD/Si is shown in Figure S2 (Supporting Information). The 45° angle view FESEM micrograph in Figure S2a (Supporting Information) depicts almost identical grains as BUNCD/Si_P_. The top view FESEM micrograph (Figure S2b, Supporting Information) shows a smooth surface with 25–40 nm clustered features. The cross‐sectional FESEM micrograph in Figure S2c (Supporting Information) shows the thickness of the deposited film to be ≈6.4 µm. The increase in surface area for BUNCD/Si_P_ can be distinctly recognized by comparing the FESEM micrographs shown for BUNCD/Si and BUNCD/Si_P_ in Figure S2 (Supporting Information) and Figure 2b, respectively. As illustrated in Figure S3 (Supporting Information), the X‐ray diffraction (XRD) spectrum of BUNCD/Si exhibits three discernible peaks at 43.9°, 75.2°, and 91.3°, which correspond to the (111), (220), and (311) planes of polycrystalline diamond, respectively. These peaks substantiate the presence of the diamond phase.^[^ ^49^, ^50^ ^]^

**Figure 4:**
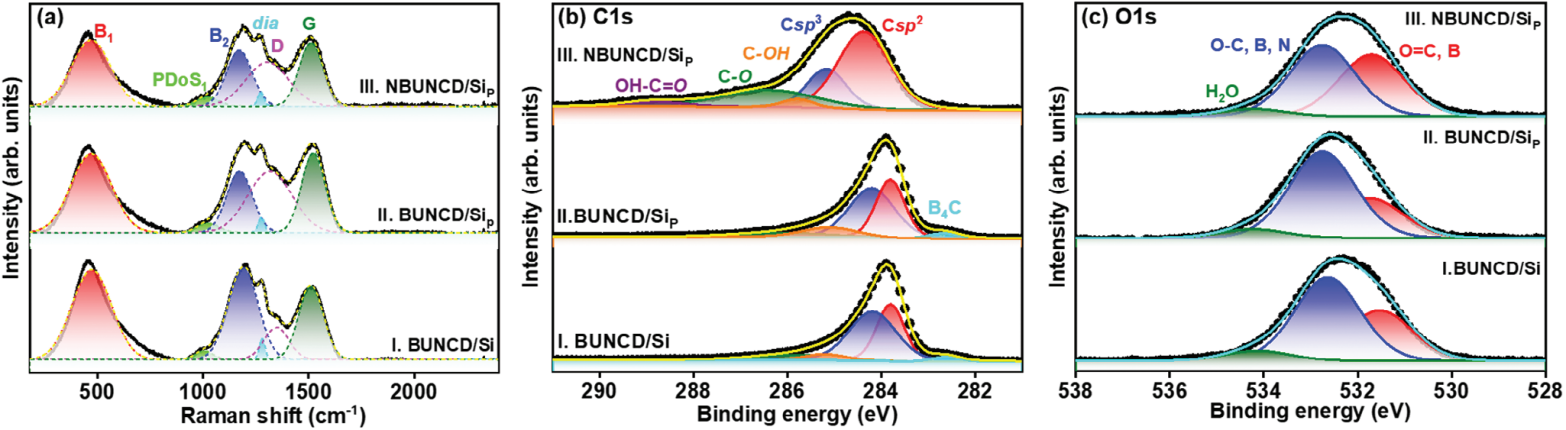
a) Raman spectra and b) XPS spectra of C1s and c) XPS spectra O1s deconvoluted peaks of BUNCD samples for I. BUNCD/Si, II. BUNCD/Si_P_, and III. NBUNCD/Si_P_.

The micro‐Raman spectrum of the NBUNCD/Si_P_ sample shown in **Figure** **4**a is deconvoluted with the Guass function multiple peaks fitting in Origin software after subtracting the background line. The sample showed a distinct, characteristic diamond peak around 1280 cm^−1^.^[^ ^51^ ^]^ The shift in the diamond peak is due to the interference between the zone‐center phonon and the continuum of electronic transitions of BUNCD occurring because of the Fano effect.^[^ ^52^ ^]^ The presence of dopant and grain boundaries in BUNCD causes a significant shifting of the peak. A series of Raman signature peak characteristics for the BUNCD samples are observed for all three BUNCD samples plotted in Figure 4a. Two broad peaks around 470 cm^−1^ (B1) and 1190 cm^−1^ (B2) are observed, attributed to the boron incorporation into the BUNCD lattice. The B1 and B2 peaks correspond to the reported maxima of phonon density of states (PDoS) and are sensitive towards the boron concentration due to the Fano effect and phonon confinement.^[^ ^53^, ^54^, ^55^ ^]^ With the incorporation of a high concentration of boron, the diamond peak (typically ≈1332 cm^−1^) suffers a drastic decrease in intensity with a significant shift to the lower wavenumber, and the relative intensity of the B1 and B2 peaks increases.^[^ ^55^ ^]^ The B1 peak is significant because it has a one‐to‐one relationship with the boron concentration in the sample. The wavenumber of the B1 peak fitted with a Lorentzian function is employed to calculate the approximate boron concentration n = 1.8 × 10^21^ in NBUNCD/Si_P_.^[^ ^47^, ^51^ ^]^ An additional minute peak is observed around 1020 cm^−1^ due to PDoS resulting from symmetry breaking in the BUNCD sample, denoted as PDoS_1_.^[^ ^54^ ^]^ The disordered carbon in the *sp^3^* cluster gives rise to a peak around 1320 cm^−1^, which is the D‐peak. The *sp^2^* carbon gives rise to the graphitization peak, G‐peak, in the BUNCD samples, observed ≈1520 cm^−1^.

X‐ray photoelectron spectroscopy (XPS) spectroscopy was employed to understand the chemical bonding and elemental content in the BUNCD samples. The C1s and O1s core level spectra fitted by the Lorentz function with the Shirley method of background correction for the BUNCD samples are shown in Figure 4b,c, respectively. The thin film samples, BUNCD/Si and BUNCD/Si_P_ (spectra I and II of Figure 4b,c), showed very similar spectra. The *sp* ^3^‐carbon peak was observed at 284.2 eV for the thin film sample, whereas the nanostructured sample was at 285.2 eV (spectrum III of Figure 4b). The shift in the *sp* ^3^‐carbon peak is due to the destruction and reconstruction of the grain boundaries after the rapid etching via the RIE process.^[^ ^56^ ^]^ In the case of NBUNCD/Si_P_, the presence of an OH─C═O peak at 288.9 eV arises due to the oxidation of the diamond during the nanostructuring process.^[^ ^57^ ^]^ The presence of *sp* ^2^‐carbon in the thin film samples is also distinctly visible at ≈283.8 eV. However, the nanostructured film possesses a slightly shifted C═C peak at 284.4 eV due to RIE. Data regarding the contents of elements in the sample calculated from the XPS spectra is shown in Table S1 (Supporting Information). The *sp* ^2^ content is higher for the case of NBUNCD/Si_P_ due to the anisotropic rapid etching via RIE.^[^ ^51^ ^]^ The presence of doped boron in the nanostructured sample appears to decrease, as seen in Figure 4b and the deconvoluted B1s spectra in Figure S4 (Supporting Information). The concentration of boron is expected to be lower in the case of NBUNCD/Si_P_ due to the re‐sputtering of the undoped amorphous carbon resulting from the RIE.^[^ ^58^ ^]^ Figure 4c displays the O1s spectra of BUNCD samples, where three distinct peaks are observed, namely, O = C, B (531.5 eV), O─C, B, N (532.8 eV) and H_2_O (534.2 eV). As can be observed from Table S1 (Supporting Information), the O═C concentration for the BUNCD sample(s) nanostructured by O_2_/CF_4_ RIE is higher than that for the non‐etched thin film sample.

Atomic force microscopy (AFM) measurements were employed to assess the root mean square (RMS) roughness of the BUNCD samples. The 8 × 8 µm^2^ AFM scan is presented in Figure S5a–d (Supporting Information). The BUNCD/Si sample exhibited an RMS roughness of 28.44 nm, attributed to the deposition of faceted grains on the planar Si substrate. In contrast, BUNCD/Si_P_ showed an increased roughness of 353.44 nm, resulting from micro‐pyramids alongside the faceted grains. However, for NBUNCD/Si_P_, accurate roughness measurement is challenging due to the wider AFM tip relative to the spacing between the nanorods. Although a value of 398.03 nm was recorded, the actual roughness is likely higher^[^ ^59^ ^]^ and, in our case, indicated from a detailed view from a scan area of 0.8 × 0.8µm^2^ and RMS of 26.49 nm (Figure S5d, Supporting Information).

**Figure 5:**
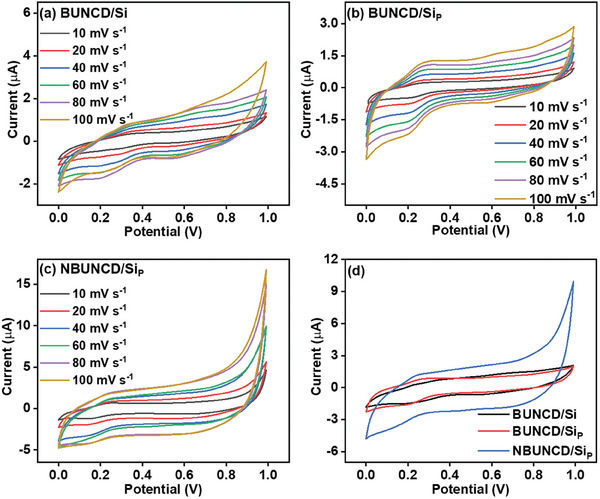
Cyclic voltammogram study in 1 m Na_2_SO_4_ at varied scan rates from 10–100 mV s^−1^ of BUNCD samples, a) BUNCD/Si, b) BUNCD/Si_P_, c) NBUNCD/Si_P_, and d) a comparison of the current response of these BUNCD samples at 60 mV s^−1^.

In order to study the EC performance of the BUNCD electrodes, cyclic voltammetry studies were carried out in the three‐electrode cell setup configuration. **Figure** **5**a–c displays the CV curves of BUNCD/Si, BUNCD/Si_P,_ and NBUNCD/Si_P_, respectively, in 1 M Na_2_SO_4_ aqueous electrolyte in the potential window from 0 to 1 V versus Ag/AgCl. The scan rates for the CV analysis varied from 10 to 100 mV s^−1^, and it was observed that the current response for all the samples increases with an increase in scan rates. A significant enhancement in the current response is observed in Figure 5c for the case of NBUNCD/Si_P_. The comparison of the CV curve of all the BUNCD samples taken at 60 mV s^−1^ scan rate is plotted in Figure 5d. Even though the current responses for the BUNCD/Si and BUNCD/Si_P_ are almost comparable, still a slight enhancement of capacitive current is resolvable for the BUNCD/Si_P_, which can be attributed to the enhancement of the surface area of a micro‐pyramidal structure. An enhancement of the area enclosed by the CV curve in the case of NBUNCD/Si_P_ is identified, which is attributed to the nanostructuring of the BUNCD film, increasing the area of interaction and the reactions of oxygen species present in NBUNCD/Si_P_ after RIE. The nanostructuring improves the number of charge transport sites by enhancing the aspect ratio of the NBUNCD/Si_P_ sample. The FESEM micrograph of NBUNCD/Si_P_ shown in Figure 2c displays available sites to adsorb and enhance the ion to transfer charge. Moreover, since the nano‐whiskers consist of disordered carbon containing *sp* ^2^‐graphitic carbon, they contribute well to the ion transfer process, thus enhancing the capacitive current of NBUNCD/Si_P_.^[^ ^60^, ^61^ ^]^ The specific areal capacitance at different scan rates for the BUNCD samples is calculated using the following Equation (1):

(1) $$C=\frac{1}{2}\;\frac{\int I\left(V\right)dV}{\Delta V\times \vartheta \times A}$$

where ∫I(V)dV is the total area or current enclosed by the CV curve, ΔV is the scanned potential window, ϑ is the scan rate under consideration, and A is the geometrical area of the working electrode. A comparative study of the specific areal capacitance calculated for the BUNCD electrodes at varied scan rates with a constant potential window is shown in Figure S6 (Supporting Information). The enhancement of the geometrical surface area in the case of BUNCD/Si_P_ enhances the specific areal capacitance compared to BUNCD/Si. Moreover, the NBUNCD/Si_P_ sample shows the highest specific areal capacitance value of 0.318 µF cm^−2^ at 10 mV s^−1^.

**Figure 6:**
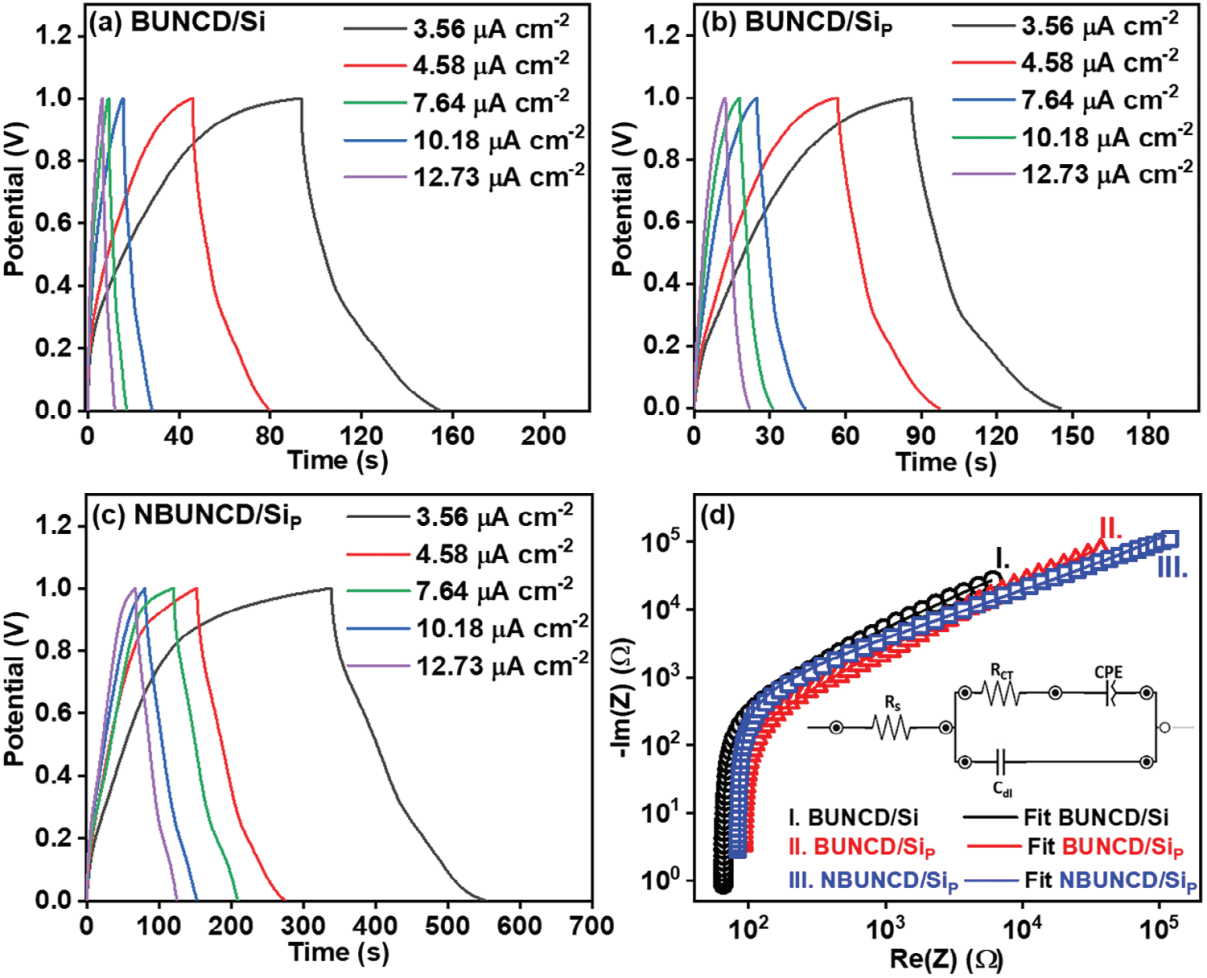
Galvanostatic charging‐discharging study with varying current densities in 1 m Na_2_SO_4_ of BUNCD samples, a) BUNCD/Si, b) BUNCD/Si_P_, c) NBUNCD/Si_P_, and d) a comparison of electrochemical impedance spectra of these BUNCD samples with the inset showing the equivalent circuit fitted to the EIS spectra for I. BUNCD/Si, II. BUNCD/Si_P_, and III. NBUNCD/Si_P_.

GCD measurements of the BUNCD samples were investigated via charging and discharging the electrodes in the predefined CV potential window at varied current densities. **Figure** **6**a–c displays the GCD curves of BUNCD/Si, BUNCD/Si_P,_ and NBUNCD/Si_P_, respectively, in 1 M Na_2_SO_4_ aqueous electrolyte in the potential window from 0 to 1 V versus Ag/AgCl recorded at current densities varied from 3.56 to 12.73 µA cm^−2^. Almost symmetrical triangle‐shaped curves are observed for the BUNCD/Si electrodes. Here, rapid charging and discharging are observed at higher ends of the current densities. The specific capacitance for the BUNCD electrodes can be calculated using Equation (2), as below:

(2) $$C=\frac{1}{2}\;\frac{I\times \Delta t}{\Delta V\times A}$$

where *I* is the current applied for GCD analysis, Δ*t* is the time taken to discharge to the minimum potential, ΔV is the scanned potential window, and A is the geometric area of the working electrode. The corresponding values of the specific capacitance calculated from the GCD curves of the BUNCD electrodes are plotted against the applied current densities and are shown in Figure S7 (Supporting Information). Here, the distinct enhanced feature of higher specific capacitance for BUNCD/Si_P_ is visible, but the highest specific capacitance is found for NBUNCD/Si_P_ of value 0.39 mF cm^−2^ at 3.56 µA cm^−2^ current density. Table S2 (Supporting Information) presents a comparison of the EC parameters of NBUNCD/Si_P_ with those of other reported nanostructured electrodes, highlighting a substantial improvement in performance over previously documented results. The NBUNCD/Si_P_ inherits higher *sp^2^* content and geometrical surface area, thus exhibiting better EDLC behavior and easy electrolyte ion transport. Thus, NBUNCD/Si_P_ is a suitable electrode for EDLC‐type applications. Figure 6d depicts a comparative plot of EIS of the BUNCD electrodes fitted with an equivalent circuit in the frequency range 0.1 Hz to 20 kHz. The equivalent circuit parameters with their corresponding values calculated from the circuit fitting are listed in **Table** **1**. The equivalent circuit consists of an equivalent series resistance (R_S_) connected in series with a network containing a charge‐transfer resistance (R_CT_) and a constant phase element (CPE) in parallel with a double‐layer capacitance (C_DL_). The NBUNCD/Si_P_ electrode demonstrates a lower R_S_ and R_CT_ value owing to its larger specific surface area. The NBUNCD/Si_P_ surface, consisting of nanorods and nano‐whiskers, provides better charge‐transfer kinetics.^[^ ^30^, ^62^, ^63^ ^]^ The clear increment of capacitance from BUNCD/Si to NBUNCD/Si_P_ is evident in Table 1. Moreover, the value of the constant n for BUNCD/Si shows a resistive nature supported by the Bode‐Bode plot in Figure S8 (Supporting Information). The phase angle (ɸ = −90°, ideal capacitor) for BUNCD/Si is −43° depicting its resistive nature, whereas for BUNCD/Si_P_ and NBUNCD/Si_P_, the phase angle is −62° and −77°, respectively.^[^ ^30^, ^63^ ^]^ The constant n for BUNCD/Si_P_ and NBUNCD/Si_P_ also increases towards 1, implying the capacitive behavior of the electrodes.^[^ ^64^ ^]^ The comparison of the values of CPE for BUNCD/Si and BUNCD/Si_P_ shows a huge enhancement from 6.9 to 15.1, which can be attributed to the formation of the micro‐pyramidal structure. However, the overall performance of NBUNCD/Si_P_ is in a relatively lower impedance range, and better capacitive behavior is observed.

**Table 1:** The equivalent circuit parameters values of BUNCD electrodes in 1 m Na_2_SO_4_ electrolyte.

Element	BUNCD/Si	BUNCD/SiP	NBUNCD/SiP	Unit
R_S_	85.8	91.1	66.2	Ω
CPE	6.9	15.1	19	µT
n	0.483	0.687	0.742	ɸ
R_ct_	0	622	591	Ω
C_DL_	1.1	3.4	18.1	µF

**Figure 7:**
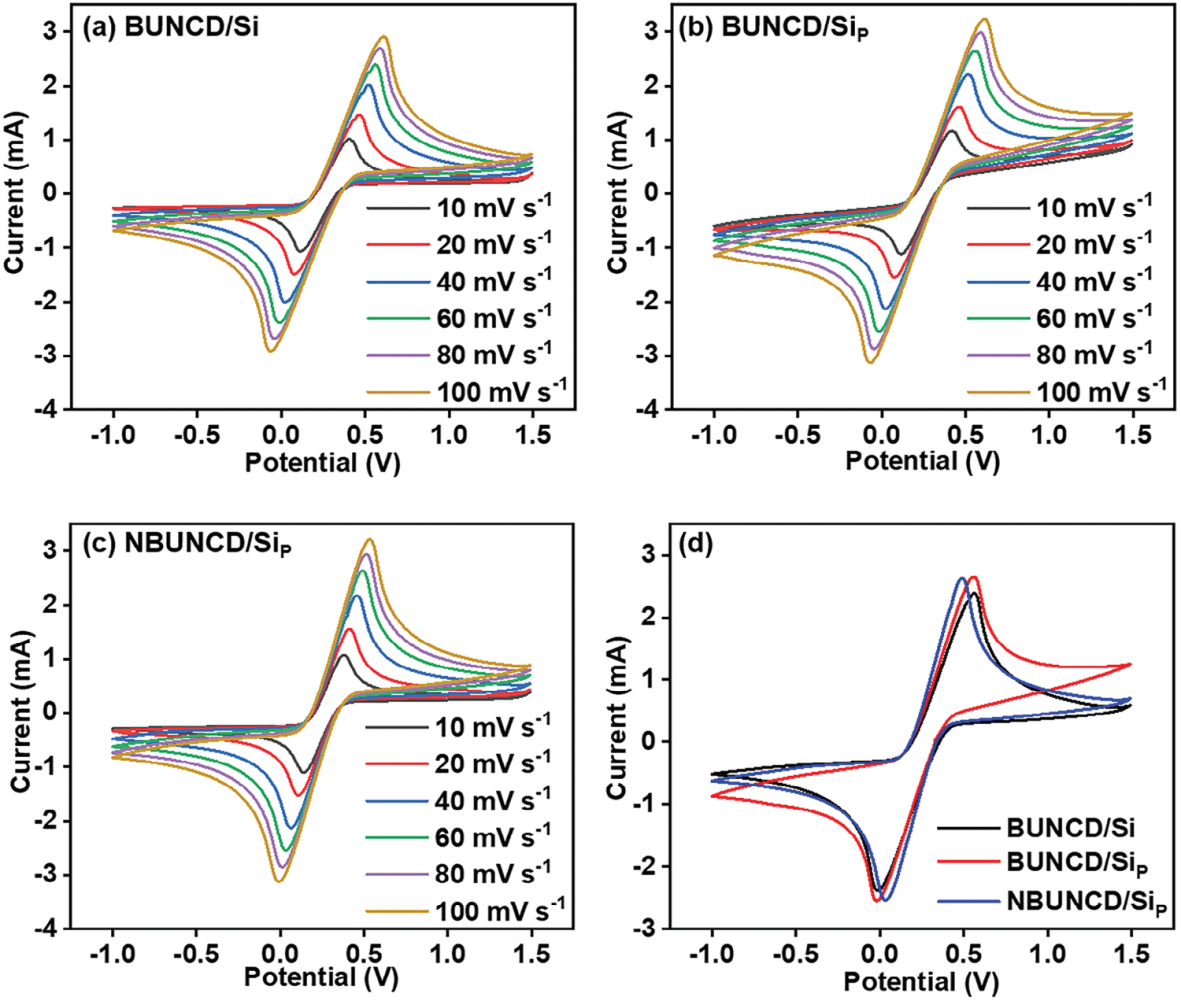
Cyclic voltammogram study in 0.05 m [Fe(CN)_6_]^3−/4−^ contained in 1 m Na_2_SO_4_ at varied scan rates from 10–100 mV s^−1^ of BUNCD samples, a) BUNCD/Si, b) BUNCD/Si_P_, c) NBUNCD/Si_P_, and d) a comparison of the current response of these BUNCD samples at 60 mV s^−1^.

The PC behavior of the BUNCD electrodes is studied by employing a redox active electrolyte in 0.05 M [Fe(CN)_6_]^3−/4−^ contained in 1 m Na_2_SO_4_ with a potential window from −1 to 1.5 V versus Ag/AgCl in a similar three‐electrode cell set up. The characteristic oxidation and reduction peaks appeared for all the BUNCD electrodes, as shown in **Figure** **7**a–c. The CV measurements are carried out at varied scan rates ranging from 10 to 100 mV s^−1^. Here, the enhancement of the area under the curve is accompanied by the enhancement of anodic‐cathodic peak separation (ΔE_P_). Figure 7d shows the overlapped plot of CV response for all three BUNCD electrodes in redox active electrolytes at 60 mV s^−1^. Figure S9 (Supporting Information) displays the variation of peak separation with the scan rates, and it is observed that only the NBUNCD/Si_P_ sample shows reversible behavior with less peak separation. In contrast, the BUNCD/Si and BUNCD/Si_P_ show an irreversible nature with a higher separation of peaks.^[^ ^65^ ^]^ The reversibility and better electron transfer kinetics observed for the case of NBUNCD/Si_P_ are assigned to *sp* ^2^‐graphitic phases and the high aspect ratio in the sample due to the RIE process. Figure S10 (Supporting Information) displays the variation of the anodic and cathodic current of the BUNCD samples plotted against the square root of the scan rates calculated from the CV curves shown in Figure 7a–c. The linear fitting of the plots suggests that the reactions in the system are diffusion‐controlled in nature.^[^ ^66^ ^]^ Furthermore, the specific areal capacitance of BUNCD film calculated from the CV curves of Figure 7a–c is shown in Figure S11 (Supporting Information). The maximum specific areal capacitance of 0.14 mA cm^−2^ at 10 mV s^−1^ is observed for the NBUNCD/Si_P_. However, it is worth noting that the BUNCD/Si_P_ sample also shows a better specific capacitance in the redox‐active electrolyte than the BUNCD/Si sample. This confirms that the formation of micro‐pyramids enhances the electrochemical performance of the BUNCD sample.

**Figure 8:**
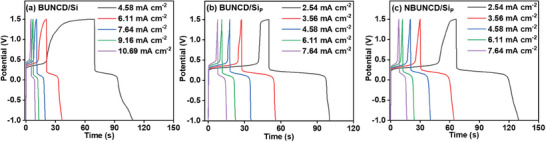
Galvanostatic charging‐discharging study with varying current densities in 0.05 m [Fe(CN)_6_]^3−/4−^ contained in 1 m Na_2_SO_4_ of BUNCD samples, a) BUNCD/Si, b) BUNCD/Si_P_, c) NBUNCD/Si_P_.

The redox‐active electrolyte was further utilized to study the charging‐discharging behavior of the BUNCD electrodes by applying varied current density at the predefined cut‐off potential window observed from the CV measurement shown in Figure 7. **Figure** **8**a–c shows a non‐linear curve for all the BUNCD electrodes with a plateau region, signifying the PC characteristics of the electrodes. This distinct feature of PC behavior is observed due to the rapid Faradaic reaction of [Fe(CN)_6_]^3−/4−^ at the electrode‐electrolyte interface. Here, the BUNCD/Si thin film sample shows a higher charging time of 70 s at a comparatively higher current of 4.58 mA cm^−2^, whereas the BUNCD/Si_P_ and NBUNCD/Si_P_ show 50 s and 66 s, respectively, at a current density of 2.54 mA cm^−2^. At 2.54 mA cm^−2^, the BUNCD/Si sample does not charge up to the desired potential. The discharge time for BUNCD/Si is 38 s, implying the non‐reversibility nature of the electrode in the redox active electrolyte. However, for BUNCD/Si_P_ and NBUNCD/Si_P_, the charging and discharging times are comparable, that is, 50 s and 64 s, respectively, implying better PC behavior. The specific capacitance of the BUNCD electrode was calculated using Equation (2) employing the GCD curves shown in Figure 8a–c and Figure S12a–c (Supporting Information), respectively. For the NBUNCD/Si_P_ sample, Figure S12c (Supporting Information) shows a maximum specific capacitance of 53.7 mF cm^−2^ at a current density of 2.54 mA cm^−2^ and the ratio of discharge time to charge time, i.e., the Coulombic efficiency (ƞ%) versus current densities for the NBUNCD/Si_P_ sample shows a 95% efficiency.

**Figure 9:**
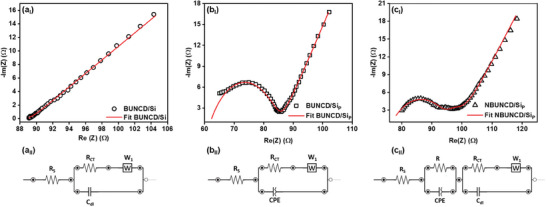
Electrochemical Impedance Spectroscopy in 0.05 m Fe(CN)_6_^3−/4−^ contained in 1 m Na_2_SO_4_ of BUNCD samples, a) BUNCD/Si, b) BUNCD/Si_P_, and c) NBUNCD/Si_P_, with (a_I_, b_I_, c_I_) EIS spectra and (a_II_, b_II_, c_II_) corresponding equivalent circuit for the EIS spectra.

**Figure** **9** shows the typical Nyquist plot for the BUNCD electrodes with the corresponding equivalent circuits fitted for the spectra in 0.05 m Fe(CN)_6_^3−/4−^ contained in 1 m Na_2_SO_4_. Figure 9a_I_ shows the EIS spectrum of BUNCD/Si with the corresponding Randles circuit containing the equivalent series resistance (R_S_), charge transfer resistance (R_CT_), diffusion resistance (W_1_), and double layer capacitance (C_DL_) as shown in Figure 9a_II_. For the Faradaic reaction taking place $\left(O+e^{-}⇋R\right)$, the charging of the electrode is occurring in parallel. Hence, R_CT_ and C_DL_ are connected in parallel. However, the rate of Faradaic reaction is controlled by the diffusion of ions to the electrode surface; hence, the circuit consists of a series connection of Warburg element (W_1_) and R_CT_.^[^ ^65^, ^67^ ^]^ Figure 9b_I_ shows the EIS spectrum of BUNCD/Si_P_ with a distinctive semicircle and the equivalent circuit similar to BUNCD/Si but consisting of a CPE instead of C_DL_. The CPE is added to model the non‐ideal capacitance behavior of the electrode arising due to the surface roughness, non‐homogeneity, and surface porosity.^[^ ^64^ ^]^ The formation of micro‐pyramids in the substrate enhances the surface area and roughness, resulting in a non‐ideal capacitance in the case of BUNCD/Si_P_. The equivalent circuit is critical for the NBUNCD/Si_P_, as shown in Figure 9c_II_. The first part of the circuit consists of the R_S_, describing the solution resistance and the internal resistance of the electrode. The second part consists of resistance arising due to the rate of redox reactions at the electrode‐electrolyte interface in parallel connection with CPE, characterizing the frequency dispersion due to the formation of nanostructures, diffusion of ions, and nature of the electrode.^[^ ^68^ ^]^ The final part consists of an R_CT_ and a C_DL_ associated with the kinetics of the electrode‐electrolyte interface. The W_1_ depicts the diffusion of ions into the porous structure of the electrode.^[^ ^66^, ^68^ ^]^ The NBUNCD/Si_P_ shows higher capacitance and less R_CT_, as shown in **Table** **2**.

**Table 2:** The equivalent circuit parameter values of BUNCD electrodes in 0.05 m [Fe(CN)_6_]^3−/4−^ in 1 m Na_2_SO_4_ electrolyte.

BUNCD/Si						
**R_S_ (Ω)**		**R_CT_ (Ω)**		**W_1_ (σ)**	**C_DL_ (µF)**	
89.1		0.37		12	425	
BUNCD/Si_P_						
**R_S_ (Ω)**	**R_CT_ (Ω)**	**W_1_ (σ)**	**CPE (µT)**		**n_1_ (ɸ)**	
61.3	24.2	13.2	140.3		0.638	
NBUNCD/Si_P_						
**R_S_ (Ω)**	**R (Ω)**	**CPE (µT)**	**n_1_ (ɸ)**	**R_CT_ (Ω)**	**W_1_ (σ)**	**C_DL_ (µF)**
75.9	20.8	244	0.549	3.3	14.9	1460

**Figure 10:**
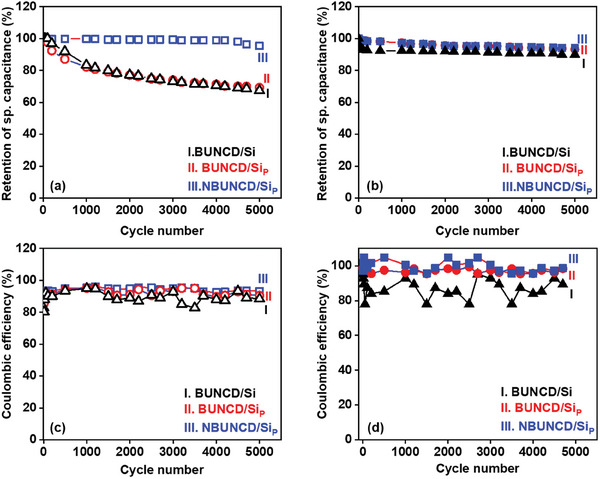
Retention of specific capacitance a,b) and Coulombic efficiency c,d) of the BUNCD samples calculated from 5000 cycles of charging−discharging in (a,c) 1 m Na_2_SO_4_ and (b,d) 0.05 m [Fe(CN)_6_]^3−/4−^ contained in 1 m Na_2_SO_4_, I. BUNCD/Si, II. BUNCD/Si_P_, and III. NBUNCD/Si_P_.

The comparative study of the specific capacitance retention and Coulombic efficiency is carried out for all the samples in both the electrolytes, as shown in **Figure** **10**. In Figure 10a, the lifecycle stability of the BUNCD samples is studied by consecutively charging and discharging the sample 5000 times in 1 m Na_2_SO_4_ aqueous electrolyte and plotting the retention of specific capacitance (ratio of final capacitance to the initial capacitance multiplied by 100) versus cycle number. It is apparent that the NBUNCD/Si_P_ has the maximum stability, and the retention is 95.49%. On the other hand, after 5000 cycles, the retention of specific capacitance falls to 67.40% and 69.26% for the BUNCD/Si and BUNCD/Si_P_, respectively. The Ragone plot for the BUNCD sample shown in Figure S13 (Supporting Information) depicts the NBUNCD/Si_P_ sample possessing a higher energy density of 54.06 µWh cm^−2^ at a power density of 0.25 µW cm^−2^. Figure 10c depicts the Coulombic efficiency variation with respect to cycle number, and the minimum recorded efficiency for the BUNCD/Si (80.35%) is lower compared to BUNCD/Si_P_ (89.71%) and NBUNCD/Si_P_ (92.53%). Similar to results in aqueous electrolytes, the NBUNCD/Si_P_ is found to have the highest retention in the redox‐active electrolyte (Figure 10b). In this case, the stability after 5000 cycles in 0.05 m [Fe(CN)_6_]^3−/4‐^ contained in 1 m Na_2_SO_4_ is 90.01%, 93.31%, and 94.05% for BUNCD/Si, BUNCD/Si_P,_ and NBUNCD/Si_P_, respectively. The Coulombic efficiency (Figure 10d) exhibits a similar trend, with the lowest efficiency recorded at 89.01% for BUNCD/Si, followed by 94.39% for BUNCD/Si_P_, and reaching 95.62% for NBUNCD/Si_P_. As observed from the impedance spectroscopy, the resistive nature of the BUNCD/Si electrode strongly agrees with the Coulombic efficiency observations.

A comparative analysis of microstructural and chemical structural modification of NBUNCD/Si_P_ before and after 5000 cycles of charging and discharging is accomplished employing FESEM and Raman spectroscopy. Figure S14a,b (Supporting Information) depicts the top‐view surface morphology of NBUNCD/Si_P_ before and after the lifecycle stability test. Figure S14a (Supporting Information) displays the nanorods with fuller circular tips, with the length of the nano‐whiskers almost comparable to that of nanorods. However, Figure S14b (Supporting Information) shows flattened tips of nanorods and suppressed nano‐whiskers. To further augment the chemical structure of the sample, Raman spectra of NBUNCD/Si_P_ before and after the lifecycle are compared and shown in Figure S14c (Supporting Information). The characteristic diamond peaks similar to Figure 4a are observed before and after samples. The variation of intensity and shifting of the peak position implies the modification in the surface chemical structure of the material as a result of the lifecycles test. Analysis of the ratio of the intensity of the D (I_D_) peak to *sp* ^3^ (I*_sp_*_3_) peak and the ratio of the intensity of *sp* ^2^ (I*_sp_*_2_) to *sp* ^3^ (I*_sp_*_3_) highlights the situation better. The I_D_/I*_sp_*_3_ ratio for the before sample is 2.66, whereas the ratio for the after sample is 2.84. There is a slight enhancement in the intensity of the D peak for the after sample, depicting the accumulation of amorphous carbon in the after sample. Moreover, the calculated value of the I*_sp2_*/I*_sp_*_3_ ratio for the after sample is 3.40, whereas for the before sample, it is 3.76. This shows the reduction of graphitic carbon content in the sample after the lifecycle measurement. These observations strongly agree with the previously reported literature.^[^ ^69^, ^70^, ^71^, ^72^ ^]^ In diamond‐graphitic composite electrodes, the charge‐transfer mediators between the electrode and electrolyte are the graphitic particles. Enache et al. have reported that the decay in the stability of the electrode material is due to the corrosion of the *sp* ^2^ graphitic active species.^[^ ^69^ ^]^

**Figure 11:**
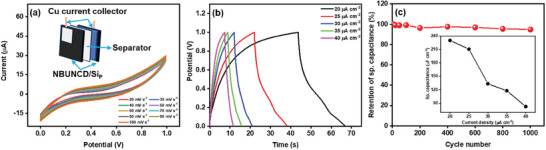
Schematic representation of the symmetric pouch cell with NBUNCD/SiP, shown in the inset of (a), along with (a) cyclic voltammetry, b) galvanostatic charge‐discharge curve, and c) life cycle stability measurement (inset showing specific capacitance calculations from (b)) of the symmetric cell using NBUNCD/SiP in 3 m KOH.

To explore the potential application of NBUNCD/Si_P_ in developing a compact energy storage device, a symmetric pouch cell was fabricated using NBUNCD/Si_P_ as the primary electrode material, with 3 m KOH serving as the electrolyte. A schematic illustration of the pouch cell is provided in the inset of **Figure** **11**a. The CV profiles of the supercapacitor at varying scan rates, ranging from 20 to 100 mV s⁻¹, within a 0–1 V potential window, are depicted in Figure 11a. The correlation between specific capacitance and scan rates is presented in Figure S15 (Supporting Information). The GCD curve, exhibiting a nearly triangular shape, confirms the EDLC behavior of the pouch cell. The variation in current densities, ranging from 20 to 40 µA cm⁻^2^, is presented in Figure 11b, while the inset of Figure 11c displays how the specific capacitance changes with respect to the applied current density. At a current density of 20 µA cm⁻^2^, the specific capacitance was recorded as 0.23 mF cm⁻^2^. The pouch cell demonstrated remarkable cycling stability, maintaining 94.83% of its initial capacitance after 1000 charge‐discharge cycles. A detailed comparison of the electrochemical performance parameters of the fabricated pouch cell with those of other reported nanostructured electrodes is provided in **Table** **3**.^[^ ^66^, ^69^, ^70^, ^71^, ^72^, ^73^ ^]^ Additionally, the NBUNCD/Si_P_ sample, with dimensions of 1 × 1 cm^2^, exhibited a maximum energy density of 31.98 µWh cm⁻^2^ and a power density of 10 µW cm⁻^2^. Notably, while the comparison table reveals deficiencies in stability and/or capacity for other nanostructured electrodes, attributed to background or side reactions occurring at the electrode surface, the chemically inert nature of diamond in the NBUNCD/Si_P_ composite ensures robust performance, further emphasizing its suitability for the EC‐SC applications.^[^ ^62^ ^]^

**Table 3:** Comparison of EC‐SC parameters of NBUNCD/Si_P_ with other reported nanostructured electrodes.

Sample	Electrolyte	Stability (number of cycles)	Sp. capacitance (current density)	Energy density	Power density	Refs.
3D symmetric MnO2/MnO2 MSCs	0.05 m Na2SO4 EMI TFSI	>80% (15 000) 80% (6000)	113 mF cm^−2^ @ 1 mA cm^−2^ 20 mF cm^−2^ @0.4 mA cm^−2^	10 µWh cm^−2^ 6.5 µWh cm^−2^	20 mW cm^−2^ 8 mW cm^−2^	[66]
RGO+CNT@CMC	PVA/H_3_PO_4_	≈100% (2000)	177 mF cm^−2^ @ 0.1 mA cm^−2^	3.84 µWh cm^−2^	20 µW cm^−2^	[69]
Hybrid carbon nanograss	0.05 m [Fe(CN)6]3−/4− in 1 m Na2SO4	95% (10 000)	0.25F cm^−2^ @ 3 mA cm^−2^	78.1−17.7 µWh cm^−2^	2.8−18.7 mW cm^−2^	[70]
LRPI	PVA‐KOH	‒	2.19 mF cm^−2^ (@20 mV/s)	1.21 µWh cm^−2^	21.9 µW cm^−2^	[71]
3D‐NSE	5 m KOH	95% (2600)	500 mF cm^−2^ (@6.4 mA cm^−2^)	385.87 µWh cm^−2^	3.82 µW cm^−2^	[72]
BDD/PPY	0.5 m H_2_SO_4_ 0.5 M TEABF_4_ (PC)	‒ ‒	1.31 mF cm^−2^ (@10 mV s^−1^) 0.42 mF cm^−2^ (@10 mV s^−1^)	1 µWh cm^−2^ 0.17 µWh cm^−2^	700 µW cm^−2^ 200 µW cm^−2^	[73]
NBUNCD/Si_P_	3 M KOH	94.8% (1000)	0.23 mF cm^−2^ (@20 µA cm^−2^)	31.98 µWh cm^−2^	10 µW cm^−2^	This work**

Higher stability and enhanced EC performance are prominently observed for NBUNCD/Si_P_. This enhancement is primarily attributed to the substrate structuring, which facilitates the formation of micro‐pyramids and the RIE process, consequently, the formation of the nanorods. These structural modifications augment the aspect ratio of the material and significantly increase the *sp* ^2^‐graphitic carbon content within the sample. Moreover, the superior high energy density and power density resulting from the substantial specific capacitance establish its suitability for applications demanding high‐performance energy storage. Overall, the synergistic effects of structural modifications and material properties position NBUNCD/Si_P_ in high‐performance EC‐SC electrodes, promising advancements in energy storage technology.

## 4 Conclusion

This study highlights the superior EC‐SC performance of NBUNCD/Si_P_. The NBUNCD/Si_P_ electrode exhibits remarkable enhancement in energy density with 54.06 µWh cm^−2^ at a power density of 0.25 µW cm^−2^. In contrast, for the thin film samples, BUNCD/Si (planar morphology) and BUNCD/Si_P_ (pyramidal morphology) exhibited lower energy densities of 15.32 µWh cm^−2^ and 14.80 µWh cm^−2^, respectively, at the same power density of 0.25 µW cm^−2^. Furthermore, NBUNCD/Si_P_ exhibits an impressive 95.5% retention of specific capacitance retention over 5000 cycles with 95% Coulombic efficiency. Comprehensive material characterization of NBUNCD/Si_P_ shows that the sample possesses a higher content of *sp* ^2^‐graphitic carbon and oxygen functional groups, contributing to enhanced electric double‐layer capacitor behavior. Moreover, the surface area of NBUNCD/Si_P_ is enhanced by the RIE nanostructures formed on the micro‐pyramidal structures. As a result, efficient pseudocapacitance properties (specific capacitance of 53.7 mF cm^−2^ at a current density of 2.54 mA cm^−2^) and stability (94.04% after 5000 cycles) are observed for the sample. These findings underline the potential of NBUNCD/Si_P_ as a promising electrode material for next‐generation EC‐SC, offering valuable guidance for future research and development in this field.

## Conflict of Interest

The authors declare no conflict of interest.

## Supporting information



Supporting Information

## Data Availability

The data that support the findings of this study are available from the corresponding author upon reasonable request.
