# Supplementary material for: Nanostructured Boron‐Doped Ultra‐Nanocrystalline Diamond Micro‐Pyramids: Efficient Electrochemical Supercapacitors
Source: Small. 2024 Dec 15;21(3):2407514. doi: 10.1002/smll.202407514 (PMC11753494; doi:10.1002/smll.202407514)
Supplement: Supplementary file 1 — Supporting Information [file SMLL-21-2407514-s001.docx]

Supporting Information

**Nanostructured Boron-Doped Ultra-nanocrystalline Diamond Micro-pyramids: Efficient Electrochemical Supercapacitors**

*Shradha Suman, Dhananjay Kumar Sharma,* *Ondrej Szabo, Benadict Rakesh, Marian Marton, Marian Vojs, Kamatchi Jothiramalingam Sankaran,^*^ and Alexander Kromka^*^*

*Shradha Suman, Benadict Rakesh, Kamatchi Jothiramalingam Sankaran*

*CSIR-Institute of Minerals and Materials Technology, Bhubaneswar 751013, India.*

*Academy of Scientific and Innovative Research (AcSIR), Ghaziabad 201002, India.*

*E-mail: kjsankaran@immt.res.in*

*Dhananjay Kumar Sharma, Ondrej Szabo, Alexander Kromka*

*Institute of Physics of the Czech Academy of Sciences, 16200 Prague, Czech Republic.*

*E-mail: kromka@fzu.cz*

*Marian Marton, Marian Vojs*

*Institute of Electronics and Photonics, Slovak University of Technology, 81219 Bratislava, Slovakia.*


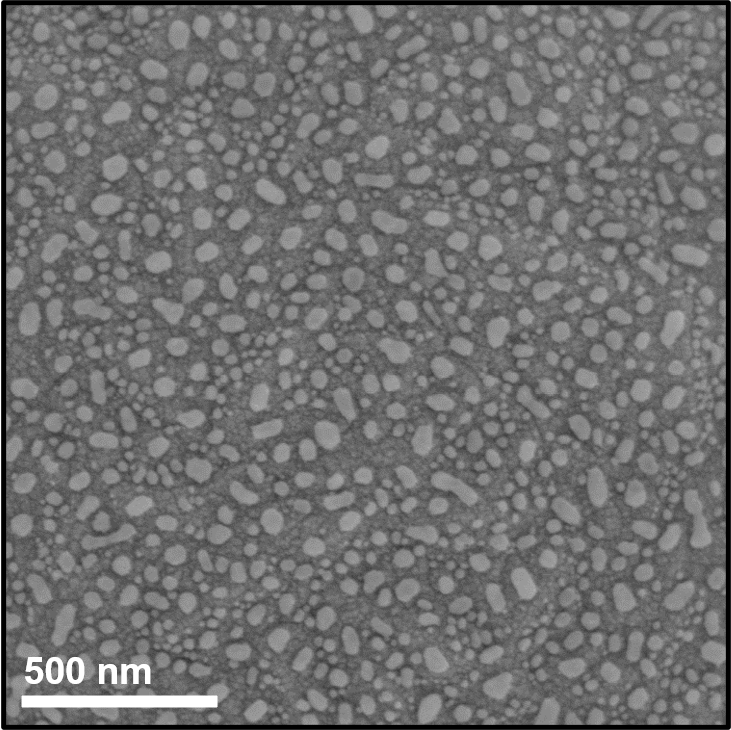


**Figure S1.** Plan-view FESEM micrographs of Au nano mask coated BUNCD/Si_P_.


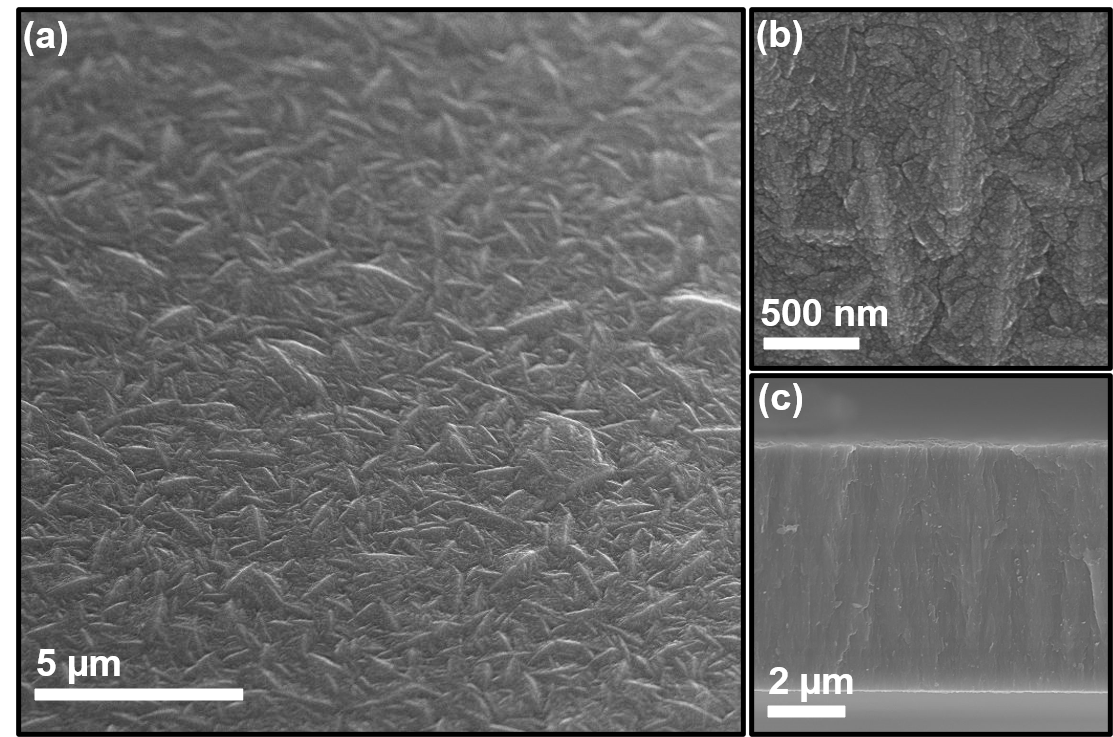


**Figure S2.** FESEM micrographs (a) 45º angle view, (b) plan-view, and (c) cross-sectional view of the BUNCD/Si.


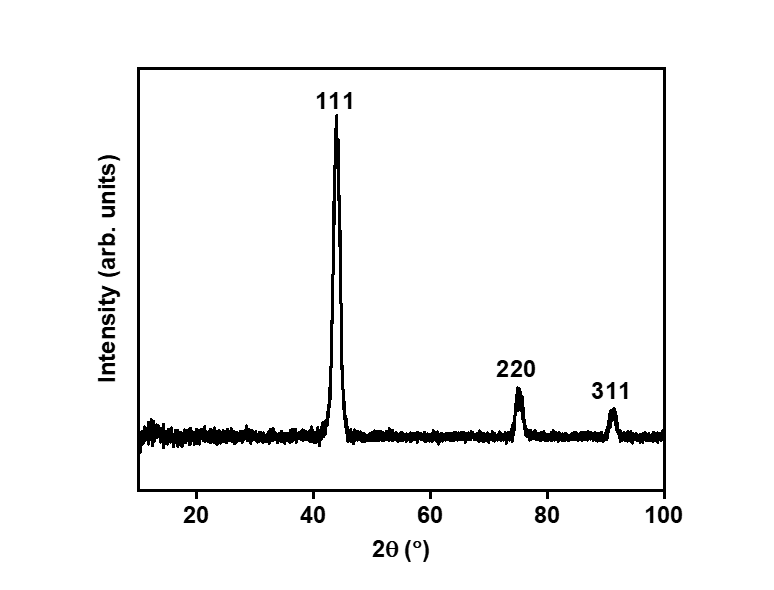


**Figure S3.** XRD spectrum of BUNCD/Si.


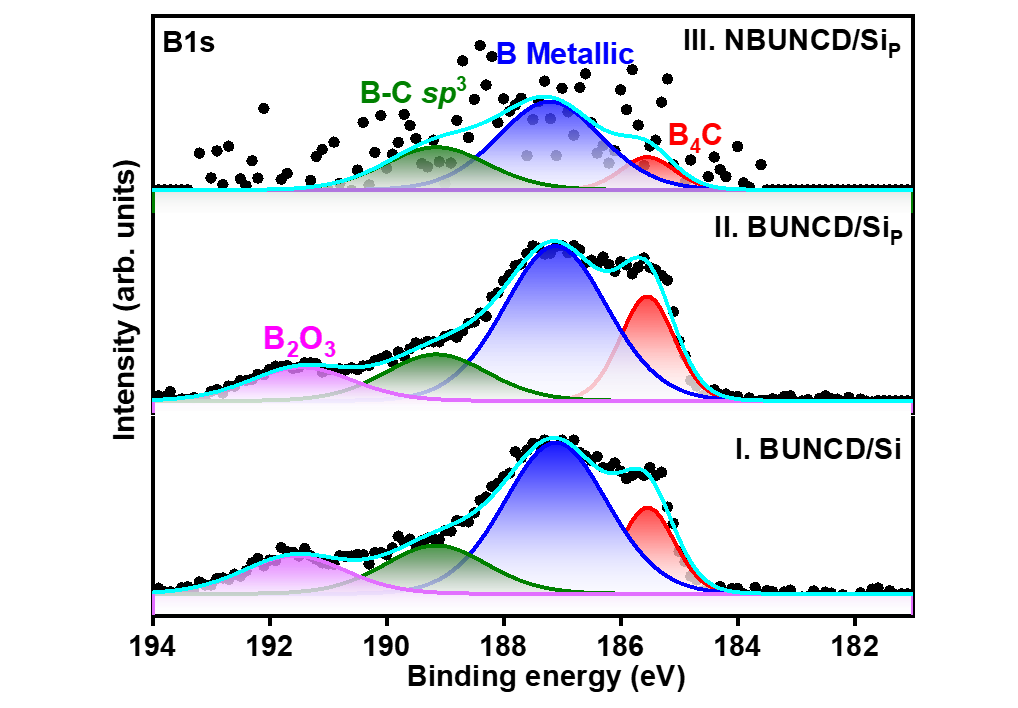


**Figure S4.** XPS spectra of B1s deconvoluted peaks of BUNCD samples with I. BUNCD/Si, II. BUNCD/Si_P_, and III. NBUNCD/Si_P_.

**Table S1**. Elemental composition of samples, determined from the XPS spectra (at%).

| **Samples** | **C1s** | | | | | | **O1s** | | | **B1s** | | | |
| --- | --- | --- | --- | --- | --- | --- | --- | --- | --- | --- | --- | --- | --- |
|  | Csp^3^ | Csp^2^ | C-O | C=O | C-O/C-OH | B_4_C | O=C, B | O-C, B, N | H2O | B_4_C | B Metallic | B-Csp^3^ | B_2_O_3_ |
| BUNCD/Si | 48.7 | 33.1 | 10.0 | - | 5.3 | 2.9 | 34.9 | 58.0 | 7.1 | 0.4 | 1.2 | 0.4 | 0.3 |
| BUNCD/Si_P_ | 44.5 | 33.2 | 7.9 |  | 10.9 | 3.4 | 29.4 | 64.0 | 6.6 | 0.4 | 1.3 | 0.4 | 0.3 |
| NBUNCD/Si_P_ | 19.7 | 52.1 | 21.1 | 3.8 | 3.3 | - | 43.7 | 50.9 | 5.4 | 0.1 | 0.4 | 0.2 | 0.0 |

**
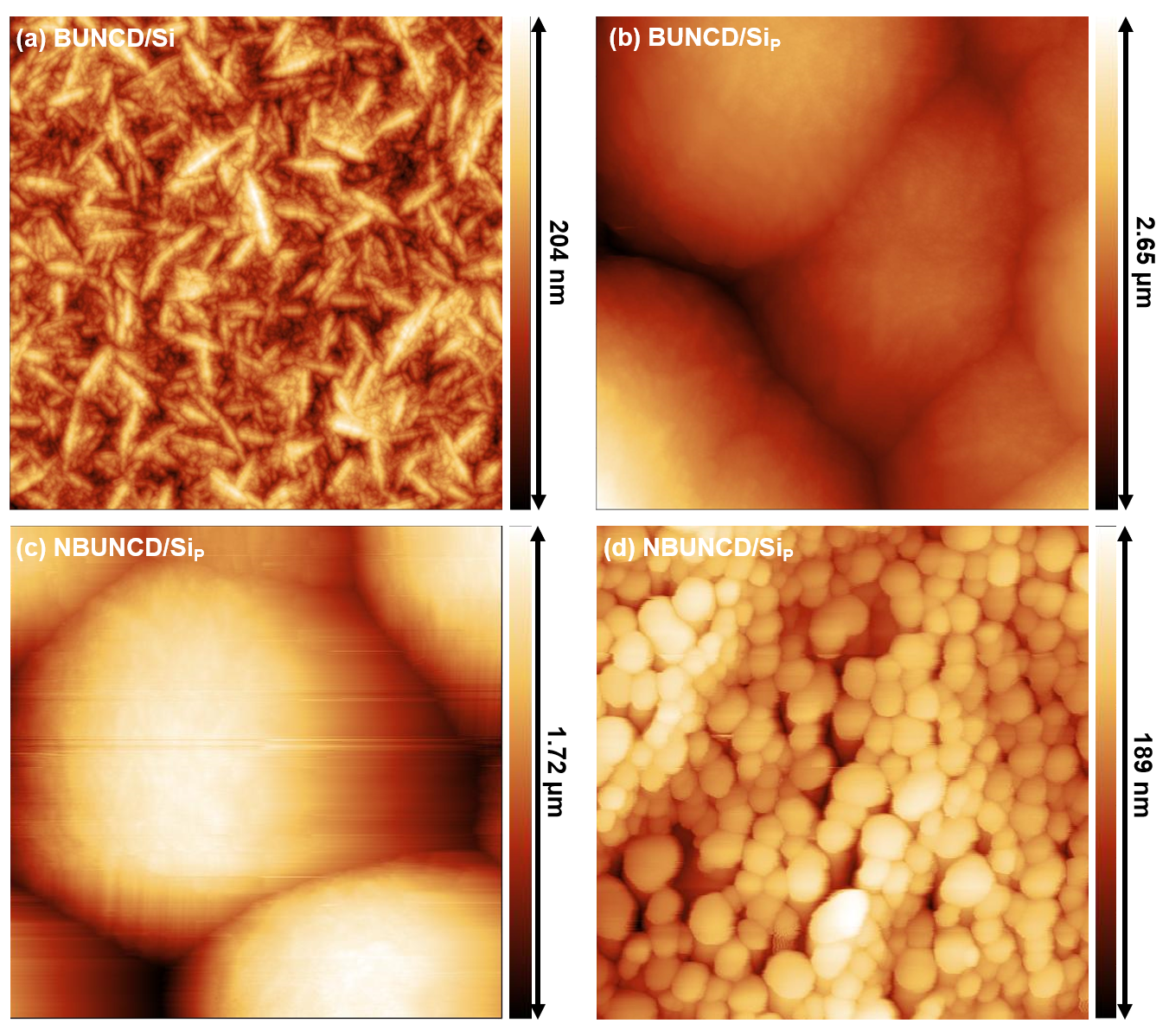
**

**Figure S5.** AFM images (8× 8µm^2^) of (a) BUNCD/Si with RMS 28.44 nm, (b) BUNCD/Si_P_ with RMS 353.44 nm, (c) NBUNCD/Si_P_ with RMS 398.03 nm and (d) detailed view (0.8× 0.8µm^2^) of NBUNCD/Si_P_ with RMS 26.5 nm.


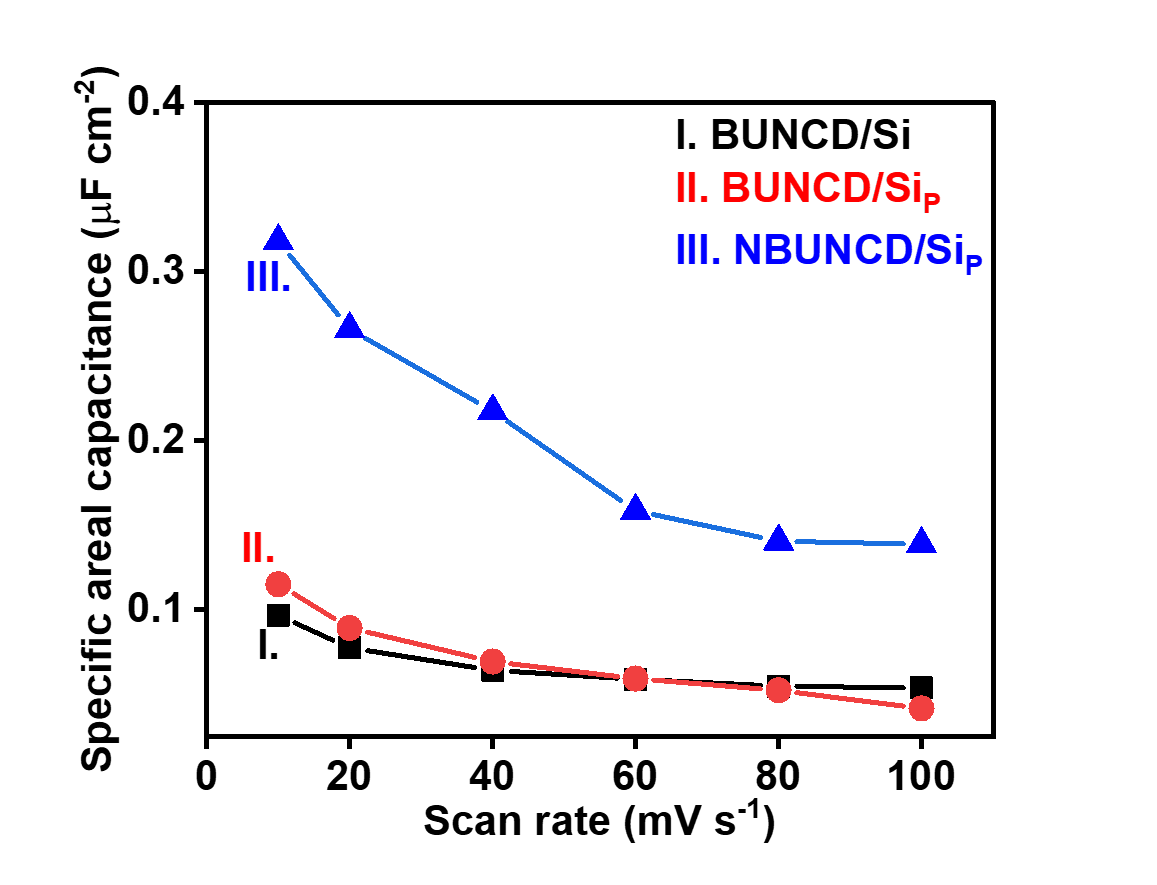


**Figure S6.** The specific areal capacitance calculated from the cyclic voltammogram study in 1M Na_2_SO_4_ with varying scan rates from 10-100 mV s^-1^ of BUNCD samples (a) BUNCD/Si, (b) BUNCD/Si_P_, and (c) NBUNCD/Si_P_, shown in Fig. 5 (a)-(c).


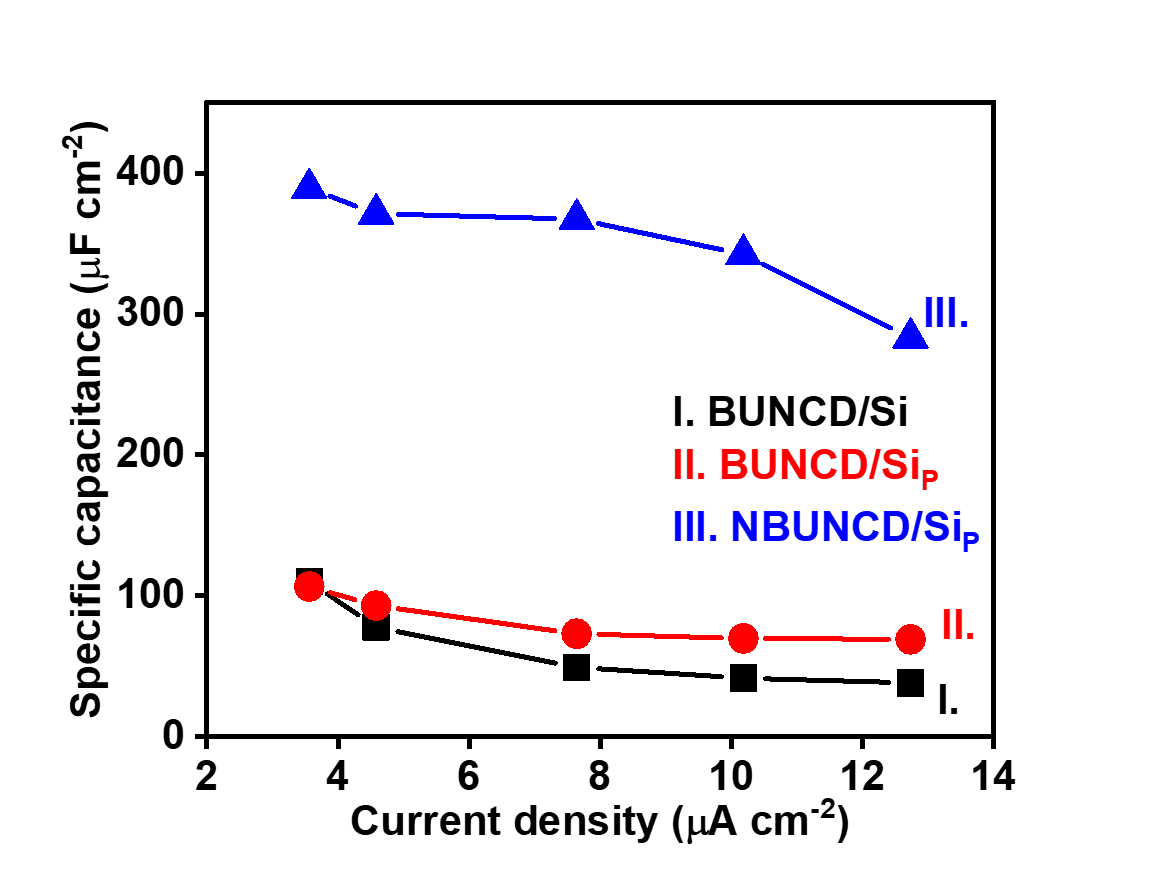


**Figure S7.** The specific capacitance calculated from the galvanostatic charging-discharging study with varying current densities in 1M Na_2_SO_4_ of BUNCD samples (a) BUNCD/Si, (b) BUNCD/Si_P_, and (c) NBUNCD/Si_P_, shown in Fig. 6 (a)-(c).

| Electrode | Electrolyte | Potential window  (V) | Specific capacitance  (mF cm^-2^) | Reference |
| --- | --- | --- | --- | --- |
| Carbon nanotubes | 1.5 M NEt_4_BF_4_ | 0 to 2.3 | 0.005-0.009 | (S1) |
| Silicon nanotrees | 1.0 M NEt_4_BF_4_ | -1.2 to -0.3 | 0.084 | (S2) |
| Graphene | TEA BF_4_ in propylene carbonate | -0.2 to 2.6 | 0.01-0.04 | (S3) |
| Single-wall carbon nanohorns | H_2_SO_4_ | 0 to 1 | 0.0064 | (S4) |
| BDD | BMIMBF_4_ | -0.2 to 0.9 | 0.01 | (S5) |
| Hybrid carbon nanograss | 1 M Na_2_SO_4_ | -0.2 to 1 | 0.3 | (S6) |
| Porous BDD | 0.5 M H_2_SO_4_ | -0.5 to 2.5 | 0.011-0.234 | (S7) |
| Diamond-coated Silicon nanowires | PMPyrrBTA | -1.1 to 1.5 | 0.317 | (S8) |
| NBUNCD/Si_P_ | 1 M Na_2_SO_4_ | 0 to 1 | 0.39 | This work** |

Table S2: Comparison of EC-SC parameters of NBUNCD/Si_P_ with other reported nanostructured electrodes.

**
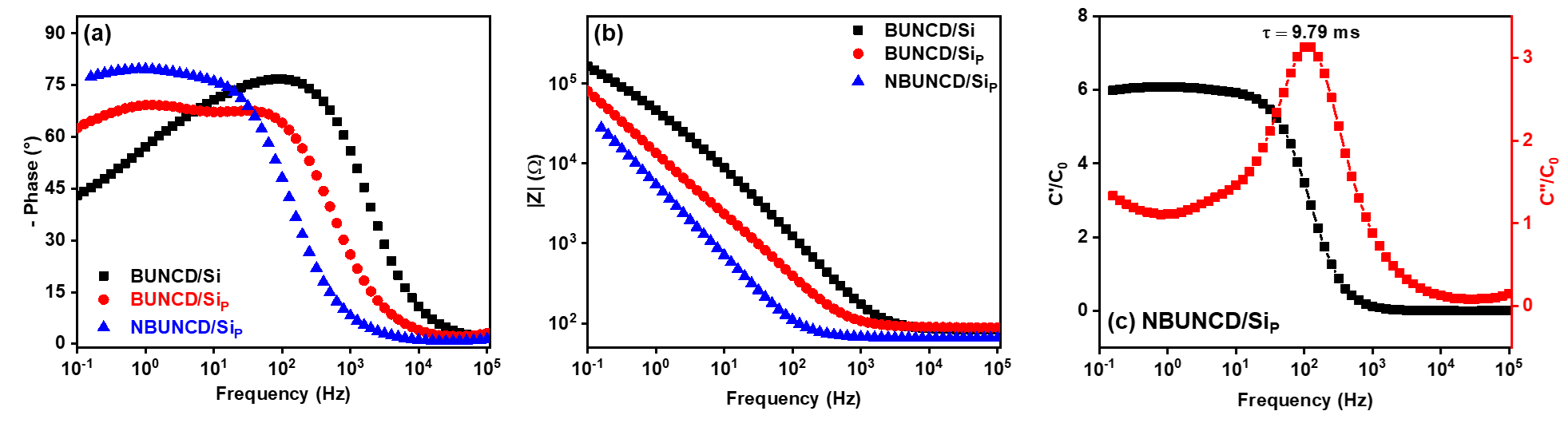
**

**Figure S8.** Bode-Bode plots for (a) phase angle, (b) impedance amplitude and (c) complex capacitance of the BUNCD samples in 1M Na_2_SO_4_.

Figure S8 presents the Bode-Bode plots, depicting the variation of phase angle and complex impedance amplitude with frequency for the BUNCD samples measured in 1M Na_2_SO_4_ electrolyte. In Figure S8a, the phase angles for the BUNCD/Si and BUNCD/Si_P_ are -43º and -62º, respectively. The BUNCD/Si shows a resistive nature, whereas BUNCD/Si_P_ shows an inclination towards capacitive behavior. This deviation from the capacitive phase angle (ɸ= -90º) suggests the presence of resistive behavior in the material.^[S9]^ The increased phase angle for the BUNCD/Si_P_ can be attributed to the higher specific surface area resulting from the formation of the micro-pyramidal structures. Conversely, the NBUNCD/Si_P_ exhibits a phase angle of -77º, indicating proximity to the capacitive behavior. The nanostructured sample demonstrates maximum capacity due to the further increase in the specific surface area due to RIE. Figure S8b shows the minimum complex impedance amplitude for the NBUNCD/Si_P_ in the entire frequency range. The impedance was employed to calculate the real ($C'(\omega)$) and imaginary ($C"(\omega)$) part of capacitance by the following equations:

$C^{'}\left( \omega\right)=\frac{-Z"(\omega)}{\omega{|Z\left( \omega\right)|}^{2}}$ (1)

$C”\left( \omega\right)=\frac{Z'(\omega)}{\omega{|Z\left( \omega\right)|}^{2}}$ (2)

where Z is the complex impedance amplitude, $Z'$ and $Z"$ are the real and imaginary parts of impedance, respectively, and $\omega$ is the angular frequency.^[S10]^ The characteristic frequency $f_{0}$ NBUNCD/Si_P_ was calculated from the imaginary part of the complex capacitance plot, as presented in Figure S8c. Therefore, the relaxation time calculated for the sample is 9.79 ms, which is comparable to the previously reported literature.^[S9–S11]^


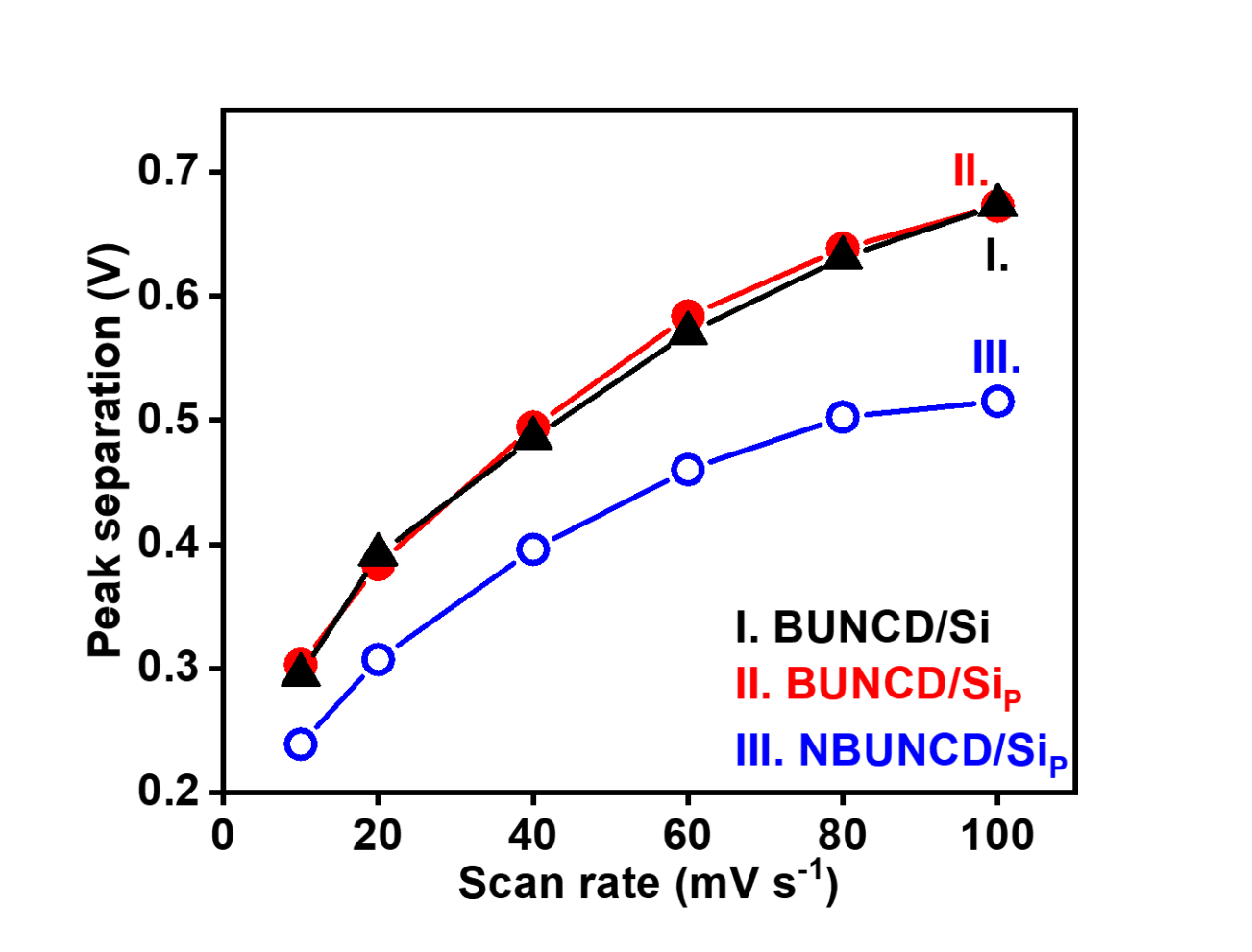


**Figure S9.** The anodic and cathodic peak separation (ΔE_P_) for BUNCD samples, I. BUNCD/Si, II. BUNCD/Si_P_, and III. NBUNCD/Si_P_, calculated from the Fig. 7 (a)-(c).


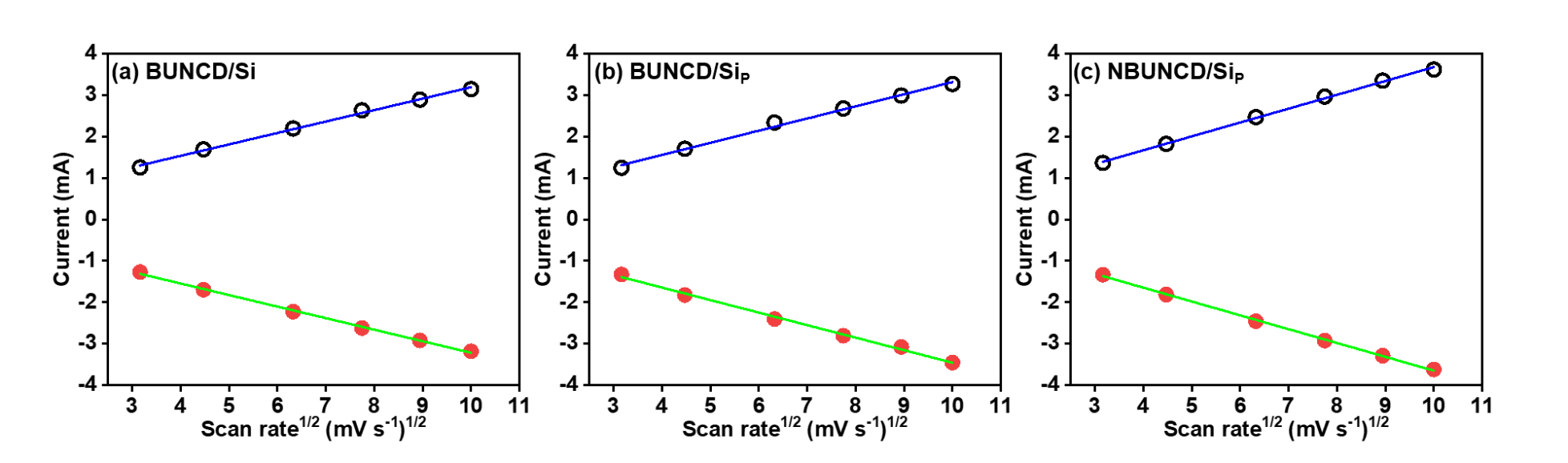


**Figure S10.** The comparison of anodic and cathodic peak current versus square root of scan rates varying from 10-100 mV s^-1^ in the 0.05 M [Fe(CN)_6_]^3-/4-^ contained in 1 M Na_2_SO_4_ electrolyte of BUNCD samples (a) BUNCD/Si, (b) BUNCD/Si_P_, and (c) NBUNCD/Si_P_, shown in Fig. 7 (a)-(c).


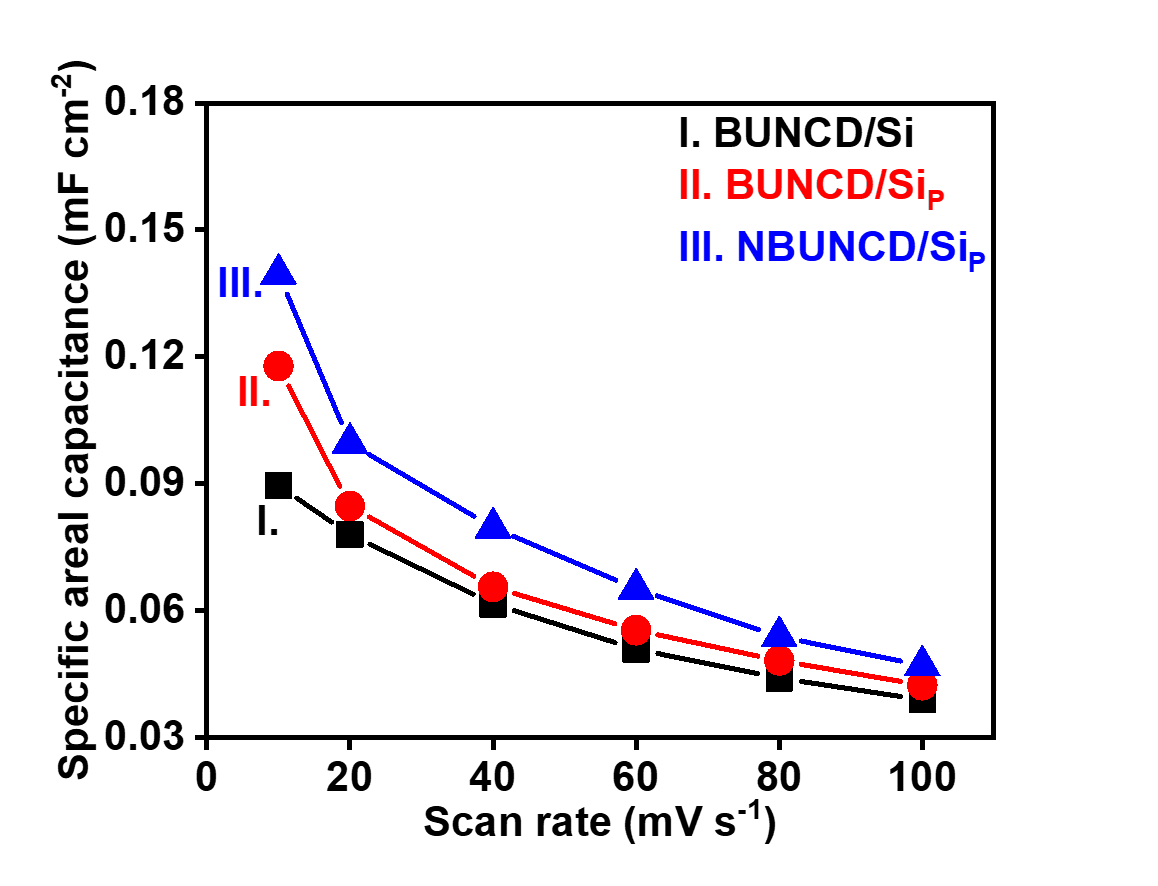


**Figure S11.** The specific areal capacitance in 0.05M [Fe(CN)_6_]^3-/4-^ contained in 1M Na_2_SO_4_ for BUNCD samples, I. BUNCD/Si, II. BUNCD/Si_P_, and III. NBUNCD/Si_P_, calculated from the Fig. 7 (a)-(c).

Figure S12 shows the specific capacitance and Coulombic efficiency for the BUCND sample in redox active electrolyte. The specific capacitance for the BUNCD/Si is observed to be higher at 60.07 mF cm^-2^ at a current density of 4.58 mA cm^-2^. However, for BUNCD/Si_P_ and NBUNCD/Si_P_, the specific capacitance is 42.69 mF cm^-2^ and 53.75 mF cm^-2^ at a current density of 2.54 mA cm^-2^. As observed in this study, the BUNCD/Si sample does not respond at a lower current density, which is a limitation for the thin film sample over a polished substrate. Similarly, the Coulombic efficiency for the BUNCD/Si sample is 56%, whereas the structured substrate samples show an efficiency higher than 95%. This implied that structuring the substrate and the diamond not only aids in enhancing the surface area but also amplifies the inherent electrochemical supercapacitor characteristics.


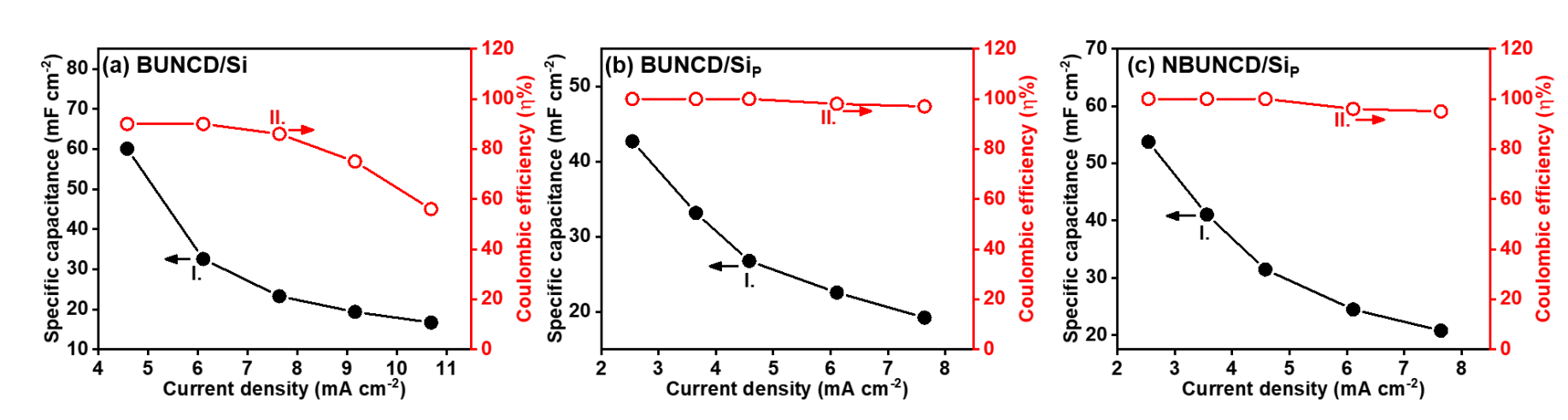


**Figure S12.** The specific capacitance and Coulombic efficiency calculated from the GCD in 0.05M [Fe(CN)_6_]^3-/4-^ contained in 1M Na_2_SO_4_ of BUNCD samples, (a) BUNCD/Si, (b) BUNCD/Si_P_, and (c) NBUNCD/Si_P_, calculated from the Fig. 8 (a)-(c).


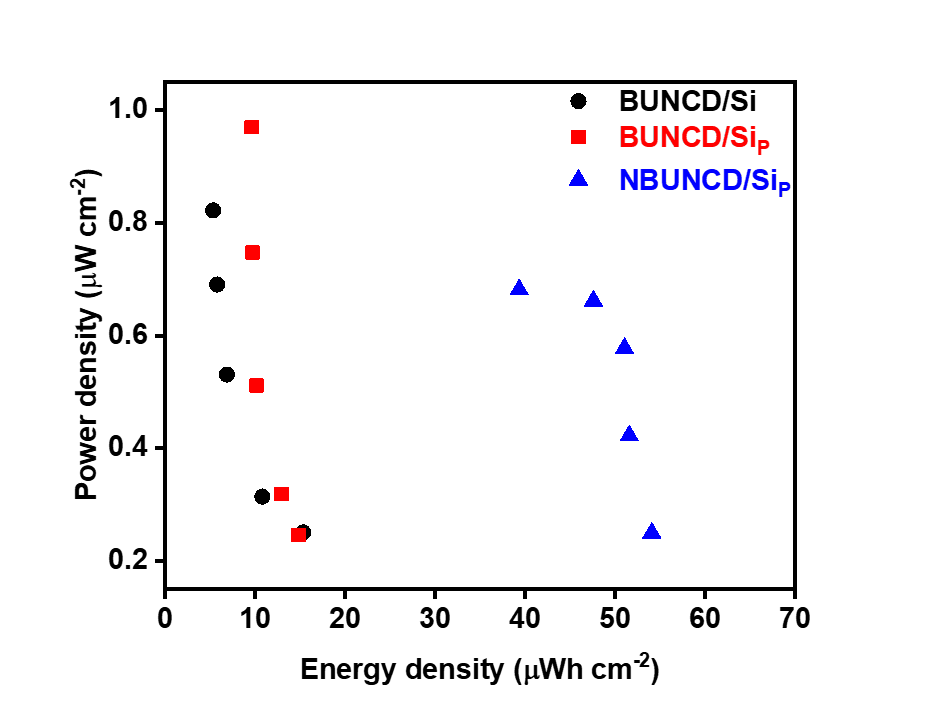


**Figure S13.** Ragone plot for BUNCD samples.

The Ragone plots for the BUNCD samples in three-electrode cell set-ups are shown in Figure S13. The energy density (E, W h cm^-2^) and power density (P, W cm^-2^) were estimated using the following equations:

$E=\frac{C \times{(\Delta V)}^{2}}{2 \times3.6}$ (1)

$P=\frac{E}{T_{d}}$ (2)

where ΔV is the scanned potential window, C is the specific capacitance calculated from the GCD curves, and T_d_ is the discharge time.^[S12, S13]^ It is apparent from the plot that the nanostructured BUNCD has a higher energy density compared to the other samples. For NBUNCD/Si_P_, the energy density and power density are observed to be 54.06 µW h cm^-2^ and 0.25 µW cm^-2^. The overall performance of NBUNCD/Si_P_ is stable with better electrochemical supercapacitor performance.


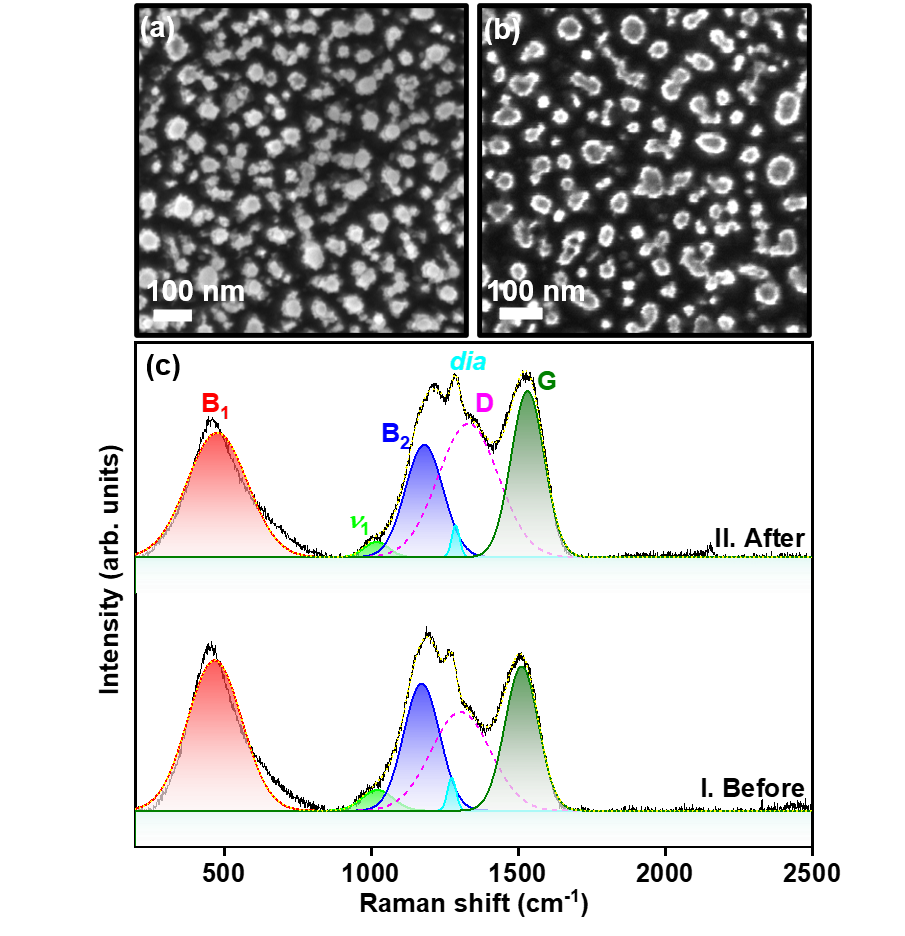


**Figure S14.** FESEM micrograph of NBUNCD/Si_P_ (a) before and (b) after 5000 charging-discharging cycles in 1M Na_2_SO_4_ electrolyte with the corresponding Raman spectra I. before and II. after the lifecycle test.

**
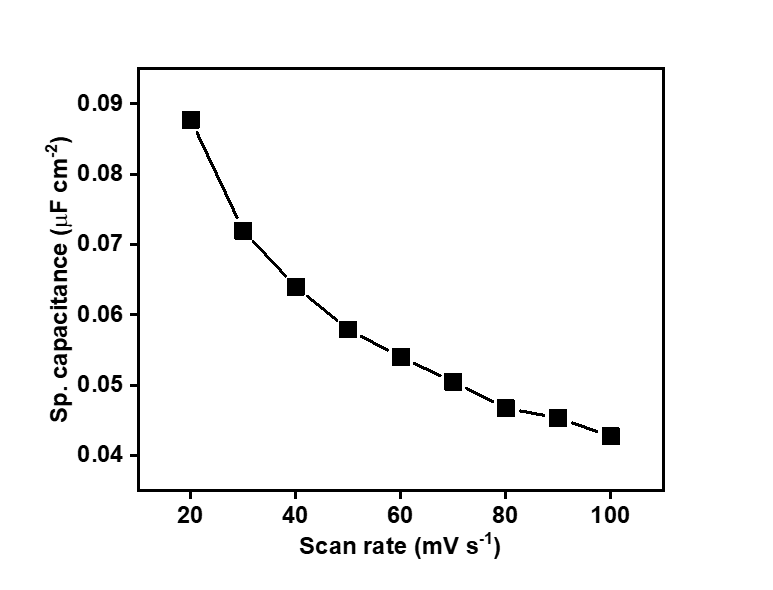
**

**Figure S15.** Variation of specific capacitance with scan rate for NBUNCD/Si_P_, calculated from the CV curve shown in Figure 11a.

**References:**

S1. C. Portet, G. Yushin, Y. Gogotsi, Carbon 45 (2007) 2511– 2518.

S2. F. Thissandier, P. Gentile, T. Brousse, G. Bidan, S. Sadki, J. Power Sources 269 (2014) 740-746.

S3. M. D. Stoller, S. Park, Y. Zhu, J. An and R. S. Ruoff, Nano Lett. 8 (2008) 3498-3502.

S4. C.-M. Yang, Y.-J. Kim, M. Endo, H. Kanoh, M. Yudasaka, S. Iijima, K. Kaneko, J. Am. Chem. Soc., 129 (2007) 20.

S5. D. Y. Kim, J. C. Yang, H. W. Kim, G. M. Swain, Electrochimica Acta 94 (2013) 49-56.

S6. D. Banerjee, K. J. Sankaran, S. Deshmukh, M. Ficek, C. Yeh, J. Ryl, I. Lin, R. Bogdanowicz, A. Kanjilal, K. Haenen, S. Sinha Roy, Nanoscale 12 (2020) 10117.

S7. T. Ohashi, J. Zhang, Y. Takasu, W. Sugimoto, Electrochim. Acta 56 (2011) 5599–5604.

S8. G. S. Gund, D. P. Dubal, D. Aradilla, W. Mueller-Sebert, G. Bidan, D. Gaboriau, P. Gentile, T. J. S. Schubert, J. Wimberg, S. Sadki, P. Gomez-Romero, 2015 International Conference on Industrial Instrumentation and Control (ICIC), Pune, India, 2015, 1125-1128.

S9. X. Wang, Y. He, Z-c Guo, H. Huang, P. Zhang, H. Lin, New Journal of Chemistry 43 (2019) 18813–18822.

S10. F. Gao, C. E. Nebel, ACS Appl Mater Interfaces 8 (2016) 28244–28254.

S11. D. Pech, M. Brunet, H Durou, P. Huang, V. Mochalin, Y. Gogotsi, P-L. Taberna, P. Simon, Nat Nanotechnol 5 (2010) 651–654.

S12. L. M. da Silva, D. A. de Lima Almeida, S. S. Oishi, A. B. Couto, N. G. Ferreira, Journal of Solid State Electrochemistry 23 (2019) 1871–1885.

S13. G. S. Gund, D. P. Dubal, N. R. Chodankar, J. Y. Cho, P. Gomez-Romero, C. Park, C. D. Lokhande, Sci Rep 5, (2015) 12454.
